# Molecular Consequences of *CCN6* Variants Encoding WISP3 in Progressive Pseudorheumatoid Dysplasia

**DOI:** 10.3390/ijms26188838

**Published:** 2025-09-11

**Authors:** Gulipek Guven Tasbicen, Ali Tufan, Batuhan Savsar, Alper Bulbul, Zeynep Tonbul, Elif Guzel, Dilay Hazal Ayhan, Ahmet Can Timucin, Umut Inci Onat, Gunseli Bayram Akcapinar, Ozlem Akgun Dogan, Yasemin Alanay, Eda Tahir Turanli

**Affiliations:** 1 Department of Medical Biotechnology, Institute of Health Sciences, Acibadem Mehmet Ali Aydinlar University, 34752 Istanbul, Turkey; gulipek.guven@acibadem.edu.tr (G.G.T.); gunseli.akcapinar@acibadem.edu.tr (G.B.A.); 2 Department of Molecular Biology and Genetics, Faculty of Engineering and Natural Sciences, Acibadem Mehmet Ali Aydinlar University, 34752 Istanbul, Turkey; ali.tufan1@live.acibadem.edu.tr (A.T.); batuhansavsar.bs@gmail.com (B.S.); elif.guzel@live.acibadem.edu.tr (E.G.); ahmet.timucin@acibadem.edu.tr (A.C.T.); inci.onat@acibadem.edu.tr (U.I.O.); 3 Department of Biostatistics and Bioinformatics, Institute of Health Sciences, Acibadem Mehmet Ali Aydinlar University, 34752 Istanbul, Turkey; abdullah.bulbul@live.acibadem.edu.tr; 4 Department of Molecular Biology and Genetics, Institute of Natural and Applied Sciences, Acibadem Mehmet Ali Aydinlar University, 34752 Istanbul, Turkey; zeynep.tonbul@acibadem.edu.tr (Z.T.); dilay.ayhan@acibadem.edu.tr (D.H.A.); 5 Department of Pediatrics, Division of Pediatric Genetics, School of Medicine, Acibadem Mehmet Ali Aydinlar University, 34752 Istanbul, Turkey; ozlem.dogan@acibadem.edu.tr; 6 Rare Diseases and Orphan Drugs Application and Research Center (ACURARE), Acibadem Mehmet Ali Aydinlar University, 34752 Istanbul, Turkey; 7 Department of Genome Studies, Institute of Health Sciences, Acibadem Mehmet Ali Aydinlar University, 34752 Istanbul, Turkey

**Keywords:** cellular communication network factor 6 (*CCN6*), WNT1-inducible signaling pathway protein 3, progressive pseudorheumatoid dysplasia, endoplasmic reticulum stress

## Abstract

Progressive pseudorheumatoid dysplasia (PPD) is a rare autosomal recessive cartilage disorder caused by biallelic variants in *CCN6*, which encodes the matricellular protein WISP3. Although WISP3 is thought to contribute to extracellular matrix (ECM) homeostasis, its precise molecular role in PPD remains unclear. To elucidate how disease-associated *CCN6* variants affect chondrocyte function, we overexpressed four variants—p.Cys52*, p.Tyr109*, p.Gly83Glu, and p.Cys114Trp—all located within the IGFBP domain, and evaluated their impact on parameters including redox balance, ER stress, ECM remodeling, gene expression, and protein–protein interactions. The p.Cys52* variant resulted in rapid degradation of WISP3, indicating a complete loss-of-function. The p.Tyr109* variant disrupted ECM regulation, markedly reducing protein interaction capacity, which was correlated with elevated mitochondrial ROS (mtROS) levels and triggered a strong response that led to programmed cell death. Although both missense variants yielded full-length proteins, their effects diverged significantly: p.Gly83Glu induced minor cellular alterations, whereas p.Cys114Trp caused severe protein destabilization, increased ROS accumulation, and high levels of ER stress. Proteomic analysis revealed that p.Cys114Trp acquired novel interaction partners, suggesting a potential gain-of-function mechanism. Collectively, these findings demonstrate that the functional consequences of *CCN6* variants depend not only on variant type or domain location but also on their positional and structural context. The distinct cellular responses elicited by each variant underscore the importance of functional validation in modeling PPD pathogenesis and offer valuable biological and therapeutic perspectives.

## 1. Introduction

Progressive pseudorheumatoid dysplasia (PPD, OMIM: 208230) is a rare autosomal recessive skeletal dysplasia that typically presents in early childhood with joint stiffness, pain, and progressive loss of mobility [1,2,3]. The condition primarily affects articular cartilage—the tissue covering the ends of bones in synovial joints—which facilitates smooth movement and absorbs mechanical loads during articulation [4]. PPD is caused by biallelic pathogenic variants in the Cellular Communication Network Factor 6 (*CCN6*) gene, located on chromosome 6q21. This gene encodes WNT1-inducible signaling pathway protein 3 (WISP3), a 354-amino-acid secreted glycoprotein predominantly expressed in chondrocytes [3,5].

The CCN family consists of six secreted matricellular proteins (CCN1–6), each containing conserved structural domains: an insulin-like growth factor binding protein (IGFBP) motif, a thrombospondin type 1 repeat (TSP1), and a cysteine knot-containing (CTCK) domain. These proteins participate in diverse biological processes by modulating key signaling pathways, including Wnt, bone morphogenetic protein (BMP), transforming growth factor beta (TGF-β), and insulin-like growth factor (IGF) [6,7,8]. *CCN6*/WISP3 plays a critical role in cartilage integrity by regulating the synthesis of type II collagen and aggrecan [3]. Loss-of-function *CCN6* variants disrupt this balance and impair chondrocyte function. Beyond its role in matrix organization, WISP3 has been implicated in mitochondrial activity, where its deficiency leads to elevated reactive oxygen species (ROS), decreased ATP levels, and impaired oxidative phosphorylation [9,10,11]. It also modulates IGF-1 signaling, limiting hypertrophy-associated stress responses [12]. Moreover, WISP3 suppresses cell migration and has tumor-suppressive effects in breast cancer, where it inhibits EMT and β-catenin signaling [13,14]. Notably, altered *CCN6* expression has also been observed in other cancers, such as colon and pancreatic cancer, where either loss or overexpression contributes to tumorigenesis, underscoring that its effects vary depending on cell type and expression level [14,15,16]. These findings highlight WISP3 as a multifunctional protein with roles extending beyond cartilage. While *CCN6* dysregulation in cancers occurs alongside other contributing factors, PPD is directly attributable to variants in *CCN6*.

Due to overlapping symptoms, PPD is frequently misdiagnosed as juvenile idiopathic arthritis (JIA) or early-onset osteoarthritis. However, unlike JIA, PPD lacks systemic inflammation; clinical markers such as erythrocyte sedimentation rate (ESR) and C-reactive protein (CRP) typically remain within normal limits [17,18]. A definitive diagnosis is established through molecular genetic testing, supported by characteristic radiographic features including platyspondyly, metaphyseal widening, and joint space narrowing [1,2]. PPD has an estimated incidence of 1 per million in the UK and seems to be more frequent in regions with high consanguinity, particularly in India, the Middle East, Eastern Mediterranean, and in Türkiye, where a founder effect has been reported for the recurrent p.Cys52X variation in these populations [2,19]. To date, more than 70 different pathogenic variants of *CCN6* have been reported, reflecting considerable allelic heterogeneity; however, consistent genotype–phenotype correlations have generally not been established [20,21]. The nonsense variant NM_198239.2:c.156C>A (p.Cys52*, rs121908901) is found in over half of Turkish PPD families [2]. In some of these families, it is co-inherited with NM_198239.2:c.248G>A (p.Gly83Glu, rs147337485), both located in exon 2. This recurrent combination suggests a possible founder effect in certain lineages [22]. Two other regionally observed variants—NM_198239.2:c.327C>A (p.Tyr109*, rs145747429) and NM_198239.2:c.342T>G (p.Cys114Trp, rs1776514016)—have been described, though their cellular consequences remain to be elucidated.

Although the variants examined in this study have been clinically reported, their functional consequences have not been systematically explored to date. However, how different *CCN6* variations influence intracellular signaling—such as Wnt, TGF-β, or mitochondrial regulation, each essential for cartilage development and skeletal stability—remains poorly defined. Gaining insight into these variant-specific mechanisms is essential to explain the cartilage-predominant manifestations observed in *CCN6*-related disorders. Despite being radiographically similar, these variants may produce divergent molecular outcomes based on variation type, affected domain, or effects on WISP3 folding, trafficking, or signaling [3,9]. Understanding whether these alterations lead to shared molecular consequences or follow separate pathways is important for clarifying genotype–phenotype relationships and identifying mechanisms that could be targeted therapeutically. While PPD variants are known, the basis of their cartilage-selective pathology remains elusive. Bridging this gap requires human chondrocyte models capable of recapitulating disease-relevant pathways.

Animal models have helped to explore *CCN6* function, but they are insufficient to successfully represent the cartilage-related phenotype observed in human PPD. This limitation makes human cell-based systems essential for studying disease-relevant mechanisms in PPD. To address cellular approaches, we first assessed the potential structural consequences of PPD-associated variants using a computational protein stability predictor. FoldX-based energy calculations targeting the IGFBP N-terminal domain of WISP3 suggested destabilizing effects for both p.Gly83Glu and p.Cys114Trp, providing a structural rationale for their functional divergence. To functionally evaluate *CCN6* variants (p.Cys52*, p.Tyr109*, p.Gly83Glu, and p.Cys114Trp), we overexpressed four alleles in human chondrocytes. This allowed us to assess their effects on extracellular matrix (ECM) dynamics, cellular stress responses, and signaling pathways relevant to ER stress and mitochondrial function.

The roles of WISP3 in tumor biology have become increasingly well defined, but its function in chondrocytes and contribution to the pathogenesis of PPD remain insufficiently understood. The present study specifically addresses this gap by investigating how disease-associated WISP3 variants alter chondrocyte function and cartilage homeostasis. We showed that nonsense and missense variants within the IGFBP domain of WISP3 exerted divergent effects on chondrocyte homeostasis, including distinct profiles of redox and ER stress responses, transcriptional remodeling, and protein–protein interactions. These findings prompted a comprehensive variant-level comparison across cellular, transcriptomic, and proteomic dimensions to elucidate potential mechanisms underlying PPD pathogenesis.

## 2. Results

### 2.1. Stability Analyses of IGFBP N-Terminal Domain Variants of WISP3:

**Figure 1:**
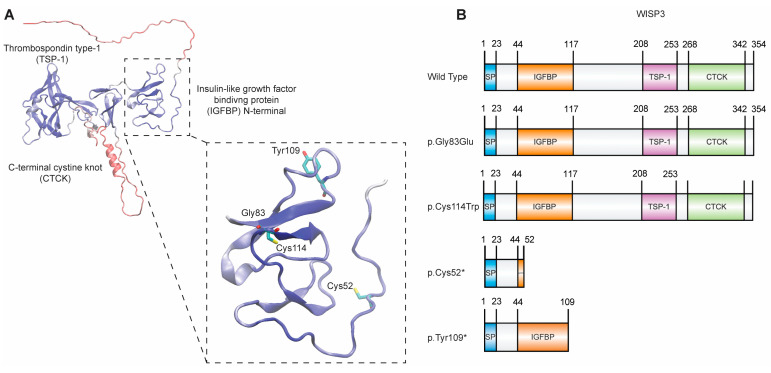
Variant positioning within the structural domains suggests differential impacts on WISP3 activity. (**A**) Structural architecture of WISP3 presented within the structure available in the AlphaFold database (AF-O95389-F1-v4). These domains (IGFBP, TSP-1, and CTCK) tend to have higher confidence scores (higher pLDDT), indicating plausible structures for FoldX-based stability analyses. The IGFBP N-terminal domain has been isolated from the whole structure (44th to 117th residues based on Uniprot ID: O95389), and the positions of some critical amino acids are shown. (**B**) Schematic overview of WISP3 domain organization in wild-type and variant forms. Missense variants p.Gly83Glu and p.Cys114Trp are indicated by vertical dashed lines and asterisks within the IGFBP domain, reflecting single amino acid substitutions without truncation. In contrast, nonsense variants p.Cys52* and p.Tyr109* result in premature stop codons, leading to loss of downstream domains to different extents.

WISP3 only has a single AlphaFold predicted structure in the literature as presented in Figure 1A. Within this structure, previously identified domains tend to have higher confidence scores in terms of structure prediction (higher predicted local distance difference test value (pLDDT)), indicating their plausibility for utilization under protein stability algorithms. For our focus in study, we isolated the Insulin-like growth factor binding protein (IGFBP) N-terminal domain out of the complete WISP3 AlphaFold predicted structure since it contains the amino acid residues that are included in the variant list used in the study (Figure 1B). Due to the fact that missense variants hold the overall structure intact we focused on non-truncation-causing, amino acid-replacing variants p.Gly83Glu and p.Cys114Trp in the computational part. Using FoldX-based stability analyses, we measured free energy change (ΔΔG) of protein stability for these variations on the isolated IGFBP N-terminal domain of WISP3 (Table 1) [23]. The results clearly demonstrated that while the p.Gly83Glu variant causes a mild destabilization on the IGFBP N-terminal domain of WISP3, the p.Cys114Trp variant could be classified potentially as a major destabilizer due to the predicted +43.33 kcal/mol ΔΔG (Table 1). This effect may be attributed to the disruption of a predicted disulfide bridge between Cys114 and Cys92, which could compromise domain stability.

### 2.2. Verification of Plasmid Constructs

The molecular consequences of PPD-associated *CCN6* variants were investigated by generating four allele-specific constructs—p.Cys52*, p.Tyr109*, p.Gly83Glu, and p.Cys114Trp—via site-directed mutagenesis of a human WISP3 expression vector (Figure 1B). A construct encoding wild-type WISP3 was also included to evaluate the effect of overexpression in human chondrocytes. Each sequence alteration was verified by Sanger sequencing (Figure S1).

**Figure 2:**
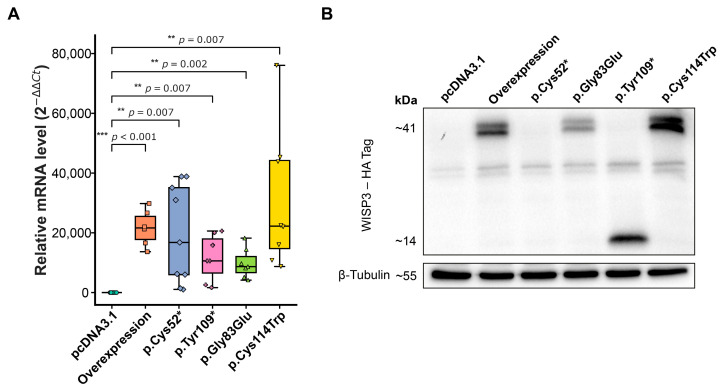
Verification of WISP3 variant expression in human chondrocytes. Human chondrocytes were transfected with plasmids encoding either wild-type WISP3 (Overexpression) or *CCN6* variants (p.Cys52*, p.Tyr109*, p.Gly83Glu, and p.Cys114Trp). (**A**) Relative *CCN6* mRNA expression levels were measured by qPCR and normalized to *ACTB*. Each condition was analyzed using four independent biological replicates, with two technical replicates per sample (*n* = 8). In box plots, the central line represents the median; box limits correspond to the 25th and 75th percentiles. Whiskers indicate the range of data, excluding outliers, which are shown as individual points. (**B**) Protein expression was assessed by Western blot using an anti-HA antibody. The full-length WISP3 protein was detected at approximately 41 kDa. β-Tubulin was used as a loading control. Statistical comparisons were performed using one-way ANOVA with Tukey’s post hoc test. Significance is represented as ** *p* < 0.01, *** *p* < 0.001.

Expression of exogenous *CCN6* was validated at both the transcript and protein levels (Figure 2). Quantitative PCR (qPCR) was performed using primers specific to the *CCN6* transcript, and relative mRNA expression was normalized to *ACTB*. All groups displayed significantly elevated *CCN6* mRNA levels compared to the empty vector control (pcDNA3.1) (Figure 2A), confirming efficient transcription and indicating that the variations did not interfere with transcription.

WISP3 protein expression was further assessed by Western blotting using an anti-HA antibody targeting the N-terminal HA tag present in all constructs. A distinct band at approximately 41 kDa, corresponding to the full-length WISP3 protein, was observed in cells transfected with the wild-type, p.Gly83Glu, and p.Cys114Trp constructs (Figure 2B). The p.Tyr109* variant produced a truncated form around 14 kDa, consistent with the presence of a premature stop codon (Figure 1B). In contrast, no detectable HA-tagged product was found in the p.Cys52*, despite preserved mRNA levels, indicating either failed translation or rapid degradation.

### 2.3. Migration and Viability of Chondrocytes Following WISP3 Variant Expression

**Figure 3:**
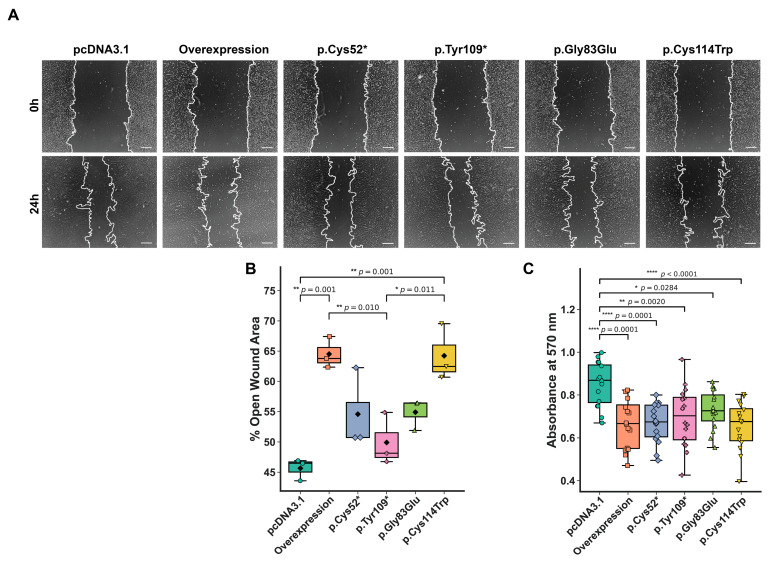
WISP3 variant expression alters wound healing capacity and cell viability in human chondrocytes. (**A**) Representative phase-contrast images from wound healing assays captured at 0 h and 24 h following scratch induction, which was performed 48 h post-transfection. Images were acquired at 4× magnification; scale bars = 250 µm (*n* = 3). (**B**) Quantification of open wound area at 24 h post-transfection, presented as a percentage of the initial wound area at 0 h. Box plots show the distribution of values; the horizontal line within each box represents the median, while the black diamonds indicate the mean. (**C**) Cell viability was evaluated 48 h post-transfection using the MTT assay. Absorbance was measured at 570 nm, and horizontal lines represent median values, which were used to compare metabolic activity across groups (*n* = 16). Statistical comparisons were performed using one-way ANOVA with Tukey’s post hoc test. Significance is represented as * *p* < 0.05, ** *p* < 0.01, **** *p* < 0.0001.

WISP3 is known to inhibit migration and the p.Cys52* variant has been shown to reduce cell viability [24]. To investigate the role of WISP3 variants in modulating cellular behavior, we performed wound healing and viability assays in transfected chondrocytes. Migration capacity was evaluated using a scratch assay, with representative phase-contrast images captured 24 h post-scratch (Figure 3A,B). Lower values correspond to greater wound closure and enhanced migratory capacity. The control group exhibited the lowest mean open wound area at 45.65%, indicating the highest migration rate. In contrast, the wild-type overexpression group displayed the largest residual wound area at 64.51%, confirming its suppressive effect on motility. The variant-expressing groups showed intermediate phenotypes, with mean open wound areas of 54.57% for p.Cys52*, 49.91% for p.Tyr109*, 54.89% for p.Gly83Glu, and 64.28% for p.Cys114Trp. These findings demonstrate *CCN*6′s variant-dependent modulation of chondrocyte migration.

Cell viability was assessed at 48 h post-transfection using the MTT assay. Absorbance at 570 nm was measured for each replicate, and the experiments were repeated eight times; the median value per group was used to represent metabolic activity, minimizing the influence of biological variation and outliers. The overexpression group exhibited the lowest metabolic activity (median = 0.667), followed by p.Cys52* (0.674) and p.Cys114Trp (0.676). The p.Tyr109* and p.Gly83Glu variants showed relatively higher activity (0.702 and 0.726, respectively), while the control group displayed the highest viability (0.868) (Figure 3C). To assess whether this reduction reflects dosage sensitivity, we transfected increasing amounts of wild-type *CCN6* plasmid, which led to a dose-dependent suppression of metabolic activity compared to control cells, as demonstrated by MTT assay results (Figure S2). According to population-level genomic constraint metrics, *CCN6* is not considered dosage-sensitive (pLI = 0.0; HI score = 30; HI% = 48.76; LOEUF = 1.055). This apparent discrepancy between cellular responses and population-based dosage sensitivity metrics suggests the requirement for further investigation to clarify context-dependent effects of *CCN6* abundance on chondrocyte viability.

### 2.4. Adhesion and Matrix Remodeling Properties of Chondrocytes Expressing WISP3 Variants

**Figure 4:**
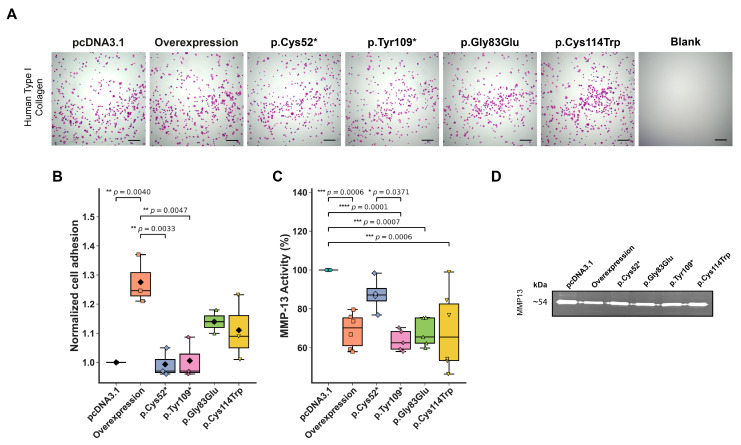
Evaluation of chondrocyte adhesion and extracellular matrix (ECM) remodeling in response to WISP3 variants. Human chondrocytes were transfected with pcDNA3.1, wild-type, p.Cys52*, p.Tyr109*, p.Gly83Glu, p.Cys114Trp. (**A**) Representative images from the cell adhesion assay performed on human type I collagen-coated wells. Cells were fixed and stained with crystal violet after seeding (scale bar = 250 μm). (**B**) Spectrophotometric quantification of attached cells based on crystal violet absorbance, normalized to control (*n* = 3). (**C**) Densitometric quantification of MMP-13 activity from zymography, presented as percentage activity normalized to the control condition (*n* = 6). (**D**) Representative zymogram showing pro-MMP-13 band intensity across experimental groups. Statistical comparisons were performed using one-way ANOVA with Tukey’s post hoc test. Significance is represented as * *p* < 0.05, ** *p* < 0.01, *** *p* < 0.001, **** *p* < 0.0001.

WISP3’s main function in chondrocytes is thought to regulate ECM features [25]. To investigate how WISP3 variants influence cell–matrix (ECM) interactions, chondrocyte adhesion capacity was assessed. Adhesion is a functional readout of ECM engagement, which plays a critical role in maintaining cartilage integrity. The influence of WISP3 variants on chondrocyte adhesion was evaluated using a collagen-coated surface assay. Cells were seeded and incubated on type I collagen-coated wells. Non-adherent cells were removed by gentle washing, and adherent cells were stained with crystal violet. After imaging adherent cells under a light microscope, the bound dye was solubilized using SDS and quantified by measuring absorbance with a microplate reader. All values were normalized to the control (set as 1.00). Overexpression consistently promoted chondrocyte adhesion to the matrix, with a mean value of 1.28 across replicates, promoting a significant increase in cell adhesion compared to the control (Figure 4A,B). In comparison, the nonsense variants p.Cys52* and p.Tyr109* exhibited adhesion levels similar to the baseline (0.99 and 1.00, respectively), signaling a loss-of-function. The missense variants, p.Gly83Glu and p.Cys114Trp, on the other hand, resulted in modest enhancements in attachment, with mean values of 1.14 and 1.11, respectively. These observations imply that WISP3 contributes to matrix attachment in chondrocytes and that this property is variably affected depending on the nature of the sequence alteration.

Matrix metalloproteinases (MMPs) are responsible for tissue remodeling and the degradation of the ECM, thereby playing a central role in maintaining ECM homeostasis [26]. The impact of *CCN6* variants on extracellular matrix turnover was assessed by evaluating MMP activity using gelatin zymography. Among the detectable gelatinases, only MMP-13 activity was consistently observed in conditioned media collected 72 h after transfection with variant or control constructs. MMP-13 levels, a key marker of matrix degradation, were used as a readout for variant-specific effects on ECM remodeling. Representative gel images revealed a marked reduction in gelatinolytic activity in plasmid-overexpressed cells compared to the control group, reinforcing the regulatory role of overexpression in extracellular matrix turnover. Band intensities were quantified with densitometric analysis and normalized to the control, which was set at 100% (Figure 4C,D). Overexpression of *CCN6* demonstrated decreased MMP-13 activity, with a median value of 70.3%. Among the variant-expressing cells, p.Cys52* exhibited the highest gelatinolytic activity at 87.2%, whereas p.Tyr109* showed a lower level at 62.6%. Both missense variants, p.Gly83Glu and p.Cys114Trp, displayed comparably reduced activity, each with a value of 65.9%, suggesting a variant-dependent impairment in matrix turnover and a potential disruption of extracellular matrix remodeling.

### 2.5. Mitochondrial Redox Imbalance, ER Stress Marker Expression, and Subcellular Localization of WISP3 Variants

**Figure 5:**
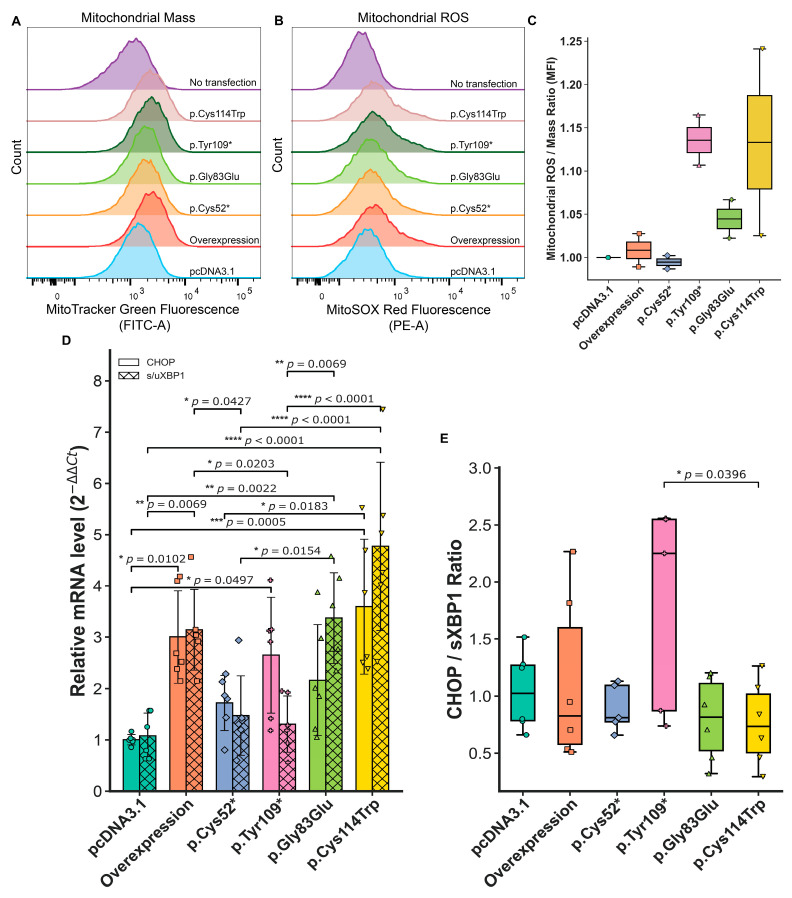
Mitochondrial ROS and ER stress marker expression upon *CCN6* variations. To investigate the impact of *CCN6* variants on mitochondrial oxidative stress and ER stress responses in chondrocytes, flow cytometry and qPCR analyses were performed. (**A**,**B**) Flow cytometry histograms showing mitochondrial mass assessed by MitoTracker Green FM (left) and mitochondrial ROS (mtROS) levels determined using MitoSOX Red staining (right) with fluorescence intensity shifts reflecting changes in mean fluorescence intensity (MFI) across conditions (*n*= 2). (**C**) Quantification of the mtROS/mass ratio, derived from MFI values and normalized to the pcDNA3.1 group (set as 1), illustrating differences in redox balance between variants. (**D**) Relative expression levels of ER stress markers *CHOP* and *s/uXBP1*, determined by qPCR. Data are represented with error bars indicating SD (*n*= 6). (**E**) *CHOP/sXBP1* expression ratio calculated as an indicator of ER stress severity (*n* = 6). Statistical comparisons were performed using one-way ANOVA with Tukey’s post hoc test. Significance is represented as * *p* < 0.05, ** *p* < 0.01, *** *p* < 0.001, **** *p* < 0.0001.

The loss of WISP3 function correlates with elevated levels of intracellular ROS and stress responses in human chondrocytes [9]. These findings suggest that *CCN6* may play a regulatory role in maintaining redox balance and mitochondrial integrity. To explore whether disease-associated WISP3 variants disrupt mitochondrial homeostasis, we assessed mitochondrial function in PPD cell culture models. Cells were stained with MitoTracker Green FM and MitoSOX Red and analyzed by flow cytometry. MitoTracker Green was used to estimate total mitochondrial content, while MitoSOX Red measured mitochondrial superoxide accumulation. Representative histogram overlays are shown in Figure 5A,B. To correct for differences in mitochondrial mass, MitoSOX mean fluorescent intensity (MFI) values were normalized to MitoTracker MFI, generating a ROS/mass ratio. This approach allows for assessing superoxide production per unit of mitochondrial content and is widely applied in studies of oxidative stress [27,28,29]. As a result, the p.Tyr109* variant showed the highest ROS/mass ratio, with a mean value of 1.14, followed by p.Cys114Trp at 1.13 and p.Gly83Glu at 1.04. The control group was used for normalization and set as 1.00, while overexpression remained nearly unchanged at 1.01. The lowest ratio was obtained in the p.Cys52*, with a value of 0.99 (Figure 5C). These findings, summarized in Figure 5C, indicate that certain WISP3 variants disproportionately elevate mitochondrial ROS relative to mitochondrial content, potentially disrupting redox homeostasis in a variant-dependent manner. Detailed flow cytometry histograms and gating strategies for all experimental conditions are provided in Supplementary Figure S3.

Endoplasmic reticulum (ER) stress has been associated with chondrocyte dysfunction in the context of genetic skeletal disorders and ECM-related protein misfolding [30,31]. To investigate whether disease-associated WISP3 variants influence this pathway, the expression of key unfolded protein response (UPR) genes was analyzed in cell culture models. In particular, two main markers were examined by qPCR: *CHOP* (C/EBP homologous protein), representing the pro-apoptotic arm of the UPR, and the spliced/unspliced *XBP1* ratio (*s/uXBP1*), indicative of the adaptive response. This approach allowed the characterization of both terminal and compensatory ER stress responses. Each condition was analyzed using three biological replicates and multiple technical replicates. However, since averaging technical replicates may not fully capture data variability, linear mixed-effects models—which account for both biological and technical replicate structure—were employed for qPCR data analysis [32].

Compared to the control group (considered as 1.00 ± 0.10-fold), the p.Cys114Trp exhibits the highest increase in both *CHOP* (3.60 ± 1.37-fold) and *s/uXBP1* (4.77 ± 1.72-fold) expression levels. Overexpression also showed elevated *CHOP* (mean: 3.00 ± 0.94-fold) and *s/uXBP1* (3.15 ± 0.82-fold) levels. In contrast, the p.Tyr109* variant showed a notable increase in *CHOP* (2.65 ± 1.18-fold) but not in *s/uXBP1* (1.30 ± 0.57-fold). The p.Gly83Glu variant induced a strong *s/uXBP1* response (3.37 ± 0.92-fold), while the *CHOP* elevation remained more modest (2.16 ± 1.13-fold). Lastly, the p.Cys52* variant was associated with relatively mild changes in both *CHOP* (1.72 ± 0.56-fold) and *s/uXBP1* (1.47 ± 0.81-fold) expression (Figure 5D). These data demonstrate that disease-associated WISP3 variants differentially modulate the expression of *CHOP* and *s/uXBP1*, indicating distinct activation patterns within the ER stress response.

The *CHOP/sXBP1* ratio was calculated as a quantitative indicator of the balance between the adaptive (*sXBP1*) and pro-apoptotic (*CHOP*) arms of the UPR [33]. A ratio above 1 indicates a predominance of pro-apoptotic signaling, whereas a ratio below 1 reflects a bias toward adaptive responses. Among all samples, the p.Tyr109* variant exhibited the highest *CHOP/sXBP1* ratio (mean: 2.63 ± 1.54-fold), suggesting impaired activation of adaptive mechanisms. In contrast, overexpression (1.13 ± 0.60-fold), p.Cys52* (1.13 ± 0.43-fold), and control (1.05 ± 0.32-fold) groups showed values close to 1, indicative of a more balanced stress response. The lowest ratios were observed in cells expressing p.Gly83Glu (0.80 ± 0.40-fold) and p.Cys114Trp (0.76 ± 0.37-fold), consistent with a predominantly adaptive stress profile (Figure 5E).

**Figure 6:**
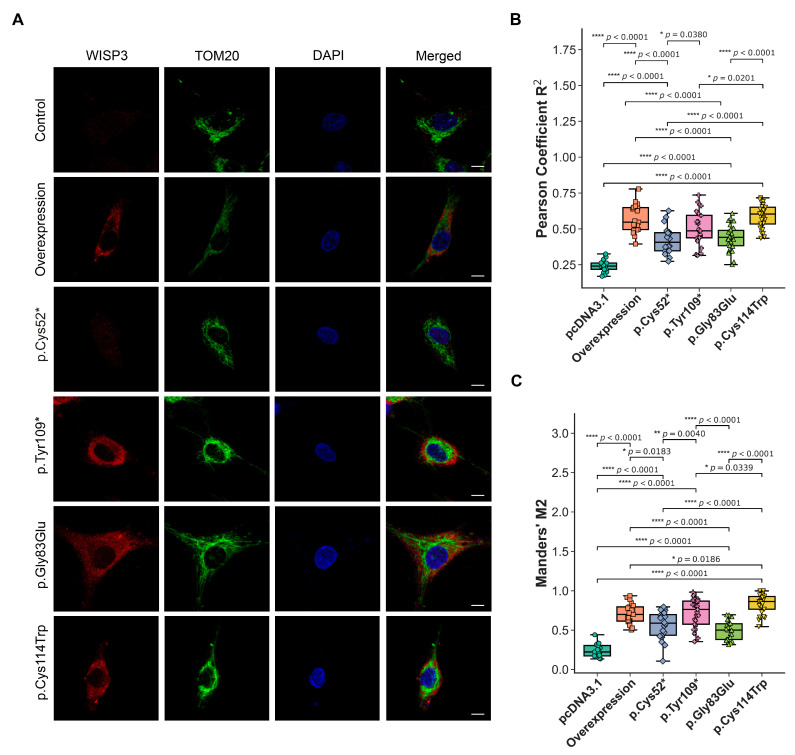
WISP3 localization and co-localization with mitochondria are differentially affected by disease-associated variants. (**A**) Representative confocal images showing subcellular localization of WISP3 (red), mitochondrial marker TOM20 (green), and nuclei (DAPI, blue) in chondrocytes transfected with either wild-type WISP3 or *CCN6* variants. Merged images indicate the extent of co-localization. Scale bar: 10 μm. (**B**) Quantification of WISP3-TOM20 co-localization using Pearson correlation coefficient (R^2^) reveals significantly reduced correlation in p.Cys52* compared to other groups. (**C**) Manders’ M2 coefficient, representing the fraction of WISP3 signal overlapping with TOM20. Each data point represents an individual cell (≥25 cells per condition from 3 biological replicates). Statistical comparisons were performed using one-way ANOVA with Tukey’s post hoc test. Significance is represented as * *p* < 0.05, ** *p* < 0.01, **** *p* < 0.0001.

The impact of disease-associated *CCN6* variants on the subcellular localization of WISP3 was evaluated by confocal co-localization analysis using the mitochondrial marker TOM20. This approach aimed at determining whether WISP3’s mitochondrial localization is altered in a variant-dependent manner. Human chondrocytes were transfected with HA-tagged wild-type or variant constructs, and co-localization was assessed by immunofluorescence staining using anti-HA (red), TOM20 (green), and DAPI (blue) (Figure 6A). Pearson correlation was used to assess the linear relationship between WISP3 and mitochondrial signals, while Manders’ M2 quantified the fraction of WISP3 signal overlapping with TOM20 [34]. Cells expressing wild-type WISP3 showed a Pearson correlation coefficient of 0.539 and a Manders’ M2 value of 0.682 (Figure 6B,C). In contrast, control cells displayed markedly lower values (Pearson = 0.269, M2 = 0.307). The p.Cys52* variant yielded values of 0.453 for Pearson coefficient and 0.666 for Manders’ M2. For p.Gly83Glu, the corresponding values were 0.496 and 0.689. The p.Tyr109* variant produced a Pearson coefficient of 0.518 and a Manders’ M2 value of 0.828. Lastly, p.Cys114Trp resulted in values of 0.562 for Pearson and 0.811 for Manders’ M2. Higher Pearson correlation coefficients indicate stronger spatial co-distribution between WISP3 and mitochondria, whereas elevated Manders’ M2 values suggest increased localization of WISP3 signal within mitochondrial regions. In this context, all variants showed enhanced mitochondrial association compared to the control, with p.Tyr109 and p.Cys114Trp demonstrating the highest overlap levels.

### 2.6. Whole Transcriptome Profiling Reveals Variant-Specific Enrichment of ER Stress, Apoptosis, and Cytoskeletal Pathways

**Figure 7:**
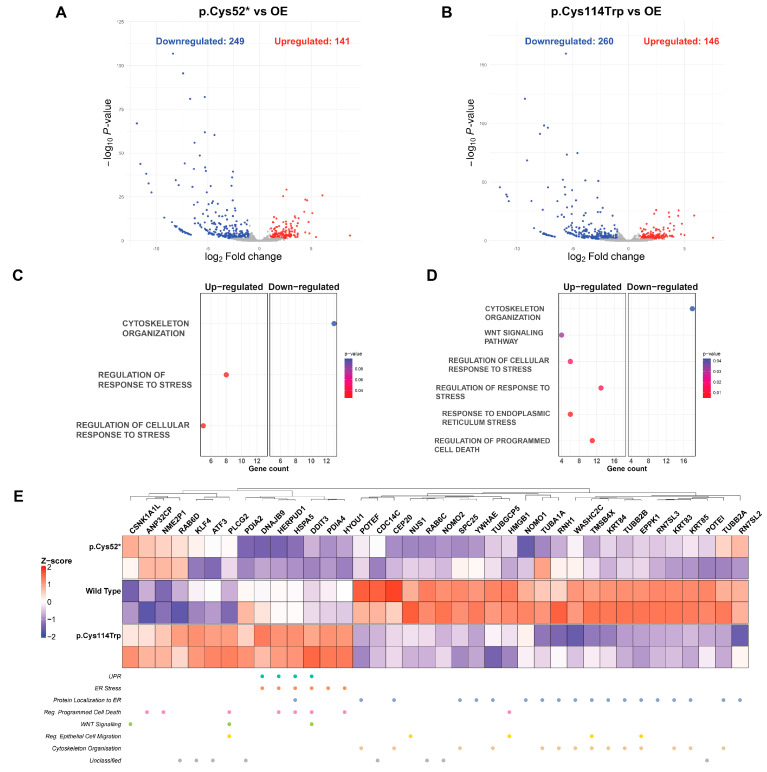
To examine the transcriptomic impact of disease-associated *CCN6* variants, human chondrocytes were transfected with wild-type WISP3 (overexpression), p.Cys52*, or p.Cys114Trp constructs, and whole-transcriptome sequencing was performed. Transcriptomic profiling of *CCN6* variants p.Cys52* and p.Cys114Trp in human chondrocytes. (*n* = 2) (**A**,**B**) Volcano plots illustrating DEGs in p.Cys52* (**A**) and p.Cys114Trp (**B**) variant-overexpressing chondrocytes compared to wild-type WISP3-overexpressing cells. Red and blue dots represent significantly upregulated and downregulated genes, respectively (adjusted *p* < 0.05, |log_2_FC| > 1). (**C**,**D**) Gene ontology (GO) enrichment analysis of significantly regulated genes in p.Cys52* (**C**) and p.Cys114Trp (**D**). Color intensity reflects statistical significance (adjusted *p*-value), and dot size corresponds to the number of genes per GO term. (**E**) Heatmap showing significantly altered gene expressions across p.Cys52*, overexpression, and p.Cys114Trp groups, with hierarchical clustering. Functional grouping of genes related to unfolded protein response, mitochondrial metabolism, and cytoskeletal organization is indicated below. Z-score represents normalized gene expression.

To investigate how nonsense or missense variants influence transcriptomic responses, RNA-seq analysis was conducted using human chondrocytes transfected with overexpression vectors encoding WT (OE), p.Cys52*, or p.Cys114Trp. Total RNA was extracted from the transfected chondrocyte cells, and libraries of two biological replicates were sequenced using the Illumina NovaSeq 6000 platform. The reads that passed quality filtering were used for the downstream analyses. Sample correlations (Figure S4A) show a high correlation coefficient between all samples (R^2^ > 0.95), signaling that no global expression shift was observed between the OE and the variants. Principal component analysis revealed distinct clustering among OE and disease-associated variant samples, indicating transcriptional divergence in pathogenic and non-pathogenic conditions (Figure S4B). The p.Cys52* variant significantly altered the expression of 390 genes (141 upregulated, 249 downregulated), while p.Cys114Trp has differentially expressed 406 genes (146 upregulated, 260 downregulated), highlighting significant gene expression shifts in each variant compared to the wild-type group (Figure 7A,B).

Biological process gene ontology (GO) enrichment analysis of these differentially expressed genes (DEGs) revealed that both variants were associated with downregulation in cytoskeleton organization, as well as upregulation in response to stress and cellular response to stress terms. Moreover, p.Cys114Trp showed pathway enrichment (*p*-value < 0.0306) in Wnt signaling (in which *CCN6* is known to function), response to ER stress, and regulation of programmed cell death (Figure 7C,D) [35]. Heatmap visualization (Figure 7E) demonstrated distinct expression patterns between wild-type and variant conditions. Genes involved in protein localization to the ER and cytoskeletal structure were predominantly upregulated in the wild-type variant. In contrast, the p.Cys114Trp variant showed elevated transcript levels associated with ER stress, unfolded protein response, and programmed cell death, distinguishing it from both wild-type and p.Cys52* variants.

Furthermore, genes such as *HSPA5*, *HERPUD1*, *DDIT3*, *KLF4*, and *PLCG2* were annotated under three or more biological processes—UPR, ER stress, Wnt signaling, and regulation of programmed cell death—and were consistently upregulated in the p.Cys114Trp condition. These findings demonstrate that WISP3 variants affect chondrocyte transcriptomes in distinct ways, with p.Cys114Trp in particular engaging cellular stress pathways more extensively than the nonsense variant. Together, these findings indicate that p.Cys114Trp-associated transcriptional changes may simultaneously affect multiple pathways rather than acting through a single regulatory axis.

### 2.7. Protein–Protein Interaction Profiling of Wild-Type and Disease-Associated WISP3

To understand the variant-dependent protein–protein interactions of WISP3, interactomes were analyzed by expressing HA-tagged wild-type, p.Tyr109*, and p.Cys114Trp constructs in chondrocyte cell lines. p.Cys114Trp variant was picked for easy comparison with RNA-seq analysis, while p.Tyr109* was picked because the other nonsense variant showed no protein expression (Figure 2B). Exogenous WISP3 was selectively isolated by anti-HA co-immunoprecipitation, and pulldown efficiency was confirmed via Western blotting and Coomassie staining. The resulting protein complexes were analyzed by liquid chromatography–tandem mass spectrometry (LC–MS/MS).

**Figure 8:**
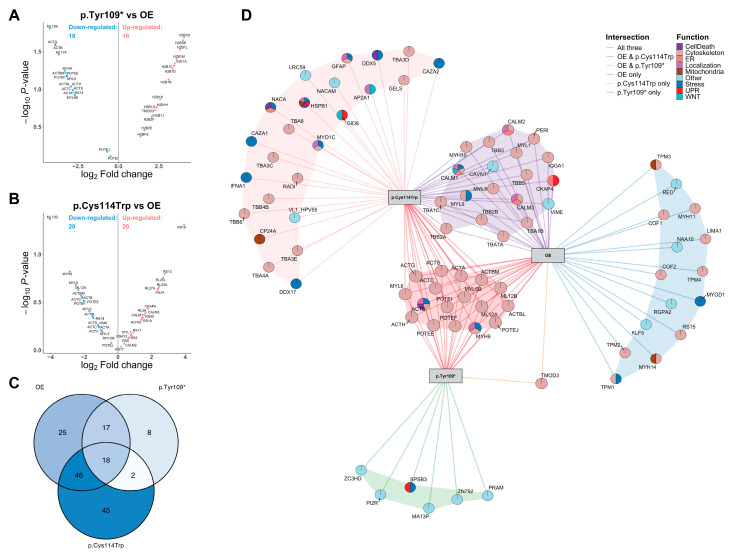
Proteomic comparison of wild-type WISP3 and variants reveals distinct interaction landscapes. (**A**,**B**) Volcano plots showing differentially enriched proteins in chondrocytes expressing p.Tyr109* (**A**) and p.Cys114Trp (**B**) compared to wild-type WISP3 (OE). Blue and red indicate significantly downregulated and upregulated proteins, respectively. (**C**) Venn diagram illustrating the number of shared and unique interactors among overexpression of WISP3 (OE), p.Tyr109*, and p.Cys114Trp conditions. (**D**) Protein–protein interaction network constructed from LC–MS/MS–based co-immunoprecipitation data. Nodes represent proteins selectively or commonly pulled down with each construct. Color coding indicates functional categories (e.g., cytoskeletal, ER-associated, mitochondrial, ribosomal, UPR-related). Interactors are grouped by their presence in one or more conditions as indicated in the legend.

Comparative proteomic analysis identified both shared and variant-specific interactors (Figure 8A–C). 18 proteins were common to all three conditions, while 35 were shared between wild-type and p.Tyr109*, 64 between wild-type and p.Cys114Trp, and 20 between the two variants. Isoform-specific interactors were also identified. In the p.Tyr109* vs. wild-type comparison, 16 proteins were upregulated and 18 downregulated; the p.Cys114Trp variant showed 20 upregulated and 20 downregulated interactors. Common LC–MS/MS contaminants such as keratins were excluded from the analysis.

Protein–protein interaction network mapping (Figure 8D; see also Figure S5 for full-scale interactome) revealed that wild-type WISP3 formed a dense and centrally organized interactome. The p.Cys114Trp variant interacted with a larger number of proteins, although these associations were reorganized into a pattern distinct from the wild-type profile, likely because of its lower protein stability. In contrast, the p.Tyr109* variant exhibited fewer interactions overall, likely reflecting the loss of key binding interfaces due to structural disruption of WISP3’s native three-dimensional conformation.

Functional classification of interactors revealed associations with cytoskeletal organization, intracellular trafficking, endoplasmic reticulum (ER)-related processes, ribosomal activity, chromatin remodeling, mitochondrial function, stress responses, the unfolded protein response (UPR), apoptosis, and WNT signaling. Cytoskeletal proteins were consistently observed across all groups, indicating partial preservation of structural associations. ER and UPR-related proteins were more frequently detected in the variant groups, whereas their representation was limited in the wild-type condition. Ribosomal proteins were commonly identified in both wild-type and p.Cys114Trp groups, while histone-associated proteins were more prominent in the p.Tyr109* variant.

Several interactors overlapped with differentially expressed transcripts from the RNA-seq dataset, including TUBB1A, TUBB2A, TUBB2B, POTEF, and POTEI. Additionally, multifunctional proteins such as CALM1–3, HSPB1, NACA, ACTB, MYH9, and CKAP4 were assigned to multiple functional categories, reflecting their involvement in both structural maintenance and stress adaptation. Collectively, these findings suggest that the p.Tyr109* variant is associated with a loss of interaction capacity, whereas the p.Cys114Trp variant promotes a broader and structurally reorganized interaction profile.

## 3. Discussion

### 3.1. Variant-Specific Destabilization of WISP3 in Chondrocytes via NMD or Structural Disruption

In this study, we functionally assessed four *CCN6* variants associated with PPD using a human chondrocyte cell culture. All variants are located within the IGFBP domain of WISP3, allowing for a direct comparison of nonsense and missense changes. The absence of detectable WISP3 in the p.Cys52* suggests either efficient targeting of its transcript by the nonsense-mediated mRNA decay (NMD) pathway or rapid post-translational degradation. In contrast, the presence of a truncated product in p.Tyr109* implies escape from NMD or reduced pathway efficiency, depending on the premature termination codons’ (PTC) context. These findings underscore that NMD activity can vary depending on the position and sequence context of the PTC [36,37,38].

The substantial destabilization predicted for the p.Cys114Trp variant aligns with its impaired functional behavior observed experimentally, as well as with its classification as pathogenic in clinical databases. In contrast, the p.Gly83Glu variant was associated with only a mild destabilizing effect. Large ΔΔG values exceeding +40 kcal/mol—such as those predicted for p.Cys114Trp—are unusual but not unprecedented. For example, β amino acid enzyme variants G165M and E391K yielded theoretical ΔΔG values of approximately +54 and +43 kcal/mol, respectively, despite FoldX’s standard error margins being only around ±0.85–1.7 kcal/mol [39,40]. Extreme ΔΔG magnitudes have also been reported in the BRCA1 BRCT domain, in which FoldX predicted ΔΔG ≈ +15.2 kcal/mol for the p.G1788V variant [41]. Classic distribution studies also top-bin FoldX ΔΔG > 14 kcal/mol, indicating such large predictions do occur, albeit rarely [42]. These previous studies could support the idea that such high values are meaningful rather than artifacts. In another benchmark study, extreme ΔΔG values were attributed to steric clashes and “hard collisions” in the mutated model, particularly for bulky substitutions [43]. In our case, Cys114 was known to interact with Cys92 through a disulfide bridge. Disruption of this bridge via insertion of a bulky tryptophan may have been penalized more that leads extreme ΔΔG value. When taken together, all these previous studies suggested that the +44 kcal/mol prediction indicated a possible catastrophic structural disruption rather than a numerical anomaly. However, since these predictions are solely based on FoldX calculations, further validation through extended molecular dynamics simulations or advanced free energy estimation approaches is required [23].

### 3.2. Truncating and Missense Variants Within the IGFBP Domain of WISP3 Impair Chondrocyte Function via Domain Loss or Disruption

Based on the viability results, elevated expression of *CCN6* may impose a metabolic burden on chondrocytes. Although previous studies have reported conflicting effects of WISP3 variants—ranging from increased proliferation to reduced cell survival—our findings suggest that the observed decline in viability is more likely related to overall *CCN6* expression levels rather than variant-specific consequences [44,45]. Nonetheless, these data raise the possibility that excessive WISP3 levels may elicit a cell type-specific stress response in chondrocytes, underscoring the need for further investigation into *CCN6* dosage effects in cartilage homeostasis [46].

Then, we evaluated how WISP3 variants influence dynamic cellular processes such as migration and matrix remodeling. Given the limited regenerative capacity of cartilage tissue, effective cell migration and wound closure are critical for maintaining matrix homeostasis and joint function. Previous studies have shown that WISP3 suppresses Wnt/β-catenin signaling and that this pathway negatively regulates cell migration [24,47]. In our study, WISP3 variants exhibited distinct effects on chondrocyte motility.

Previous studies have also suggested that WISP3, upon secretion, may act as a ligand transmitting autocrine or paracrine signals, interacting with IGF-1 via its IGFBP domain and suppressing Wnt/β-catenin signaling [11]. These mechanisms have been implicated in the regulation of cartilage structure. Since our findings suggest that Wnt/β-catenin signaling may also play a role in matrix remodeling, we assessed active MMP levels. Pro-MMP-13 levels were reduced under wild-type WISP3 overexpression, reflecting a potential protective mechanism against matrix degradation. Among the variants, p.Cys52*, which does not produce a functional protein, showed the highest pro-MMP-13 levels, while the other variants exerted a stronger suppressive effect compared to the wild-type variant. These findings raise the possibility that excessive inhibition of MMP-13 might impair physiological matrix renewal. The exclusive detection of the inactive pro-form in our cell model suggests that these differences occur at the transcriptional level. Our data are consistent with previous studies reporting reduced extracellular MMP-13 levels in PPD articular cartilage. Accordingly, our results support that WISP3 dysfunction alters matrix homeostasis through suppression of MMP-13 activity [48]. Additionally, the absence of MMP-2 and MMP-9 activity in the zymography analysis further supports the lack of an inflammatory response in PPD.

Under non-inflammatory conditions, MMP-13 transcription is primarily regulated by homeostatic signaling pathways such as Wnt/β-catenin and TGF-β/SMAD3 [17,18,47,49,50,51,52,53]. The observed changes in ECM homeostasis support the hypothesis that WISP3 may coordinate these processes through Wnt/β-catenin modulation. Although alternative pathways such as FAK and MAPK/ERK are known to contribute to migration, their potential crosstalk with Wnt signaling highlights the need to further investigate how these routes might modulate WISP3-associated signaling networks [54,55,56,57].

WISP3 is also thought to influence cartilage homeostasis by regulating chondrocyte adhesion to ECM components. In adhesion assays, cells expressing full-length WISP3 adhered more strongly to collagen-coated surfaces compared to the control group. In contrast, cells expressing nonsense variants showed reduced adhesion, likely due to the absence of the TSP1 and CTCK domains, which mediate interactions with various ECM components such as collagens, fibronectin, laminin, and integrins [5,58]. The reduced adhesion observed in cells expressing missense variants suggests that even subtle structural alterations can impair matrix engagement. These findings indicate that WISP3 supports chondrocyte–matrix interactions and highlight the importance of domain-level structural integrity in maintaining effective adhesion.

### 3.3. WISP3 Variants Differentially Disrupt ER Stress Responses and Mitochondrial Redox Homeostasis in Chondrocytes

Reactive oxygen species (ROS) are key regulators of chondrocyte homeostasis, and their accumulation contributes to cartilage degeneration in various skeletal pathologies [59]. Reduced endogenous WISP3 expression has been associated with elevated levels of both cellular and mitochondrial ROS. Additionally, nonsense variants have been shown to induce significantly higher cellular ROS levels compared to cells expressing wild-type WISP3 [9,10]. In this study, we assessed mitochondrial-specific superoxide levels instead of total ROS, thereby achieving higher organelle-level specificity. Although not statistically significant, the increased ROS/mass ratios observed in the p.Tyr109* and p.Cys114Trp variants suggest that these variants may disrupt redox homeostasis and elevate cellular stress. Mitochondria-derived excess ROS is known to impair mitochondrial structures and compromise cellular function [60]. To determine whether the observed increase in ROS/mass reflects true mitochondrial dysfunction, future analyses should evaluate parameters such as mitochondrial membrane potential (Δψ), ATP production, and mitochondrial network integrity [61].

Given the association between mitochondrial dysfunction and endoplasmic reticulum (ER) stress, we investigated whether *CCN6* variants affect ER homeostasis in chondrocytes. ER stress arises from protein misfolding and activates the unfolded protein response (UPR), a cellular mechanism implicated in the pathogenesis of several skeletal dysplasias, including chondrodysplasias [31,62,63]. Accordingly, we analyzed *CHOP* and the *s/uXBP1* expression levels, which represent the pro-apoptotic and adaptive branches of the UPR, in chondrocytes expressing disease-associated WISP3 variants, respectively.

WISP3 variants modulated the direction and magnitude of the UPR in chondrocytes in a variant-specific manner. The missense variants p.Gly83Glu and p.Cys114Trp were associated with elevated *s/uXBP1* levels, indicating activation of adaptive responses. However, the p.Cys114Trp variant also induced a marked increase in *CHOP* expression, suggesting concurrent activation of both adaptive and pro-apoptotic branches of the UPR. In contrast, the nonsense variant p.Tyr109* exhibited high *CHOP* levels, indicating a predominantly pro-apoptotic response. This distinction was further supported by *CHOP/sXBP1* ratio analysis: while the ratio remained below 1 in the missense variants, consistent with an adaptive response, it was markedly elevated in p.Tyr109*, reflecting insufficient compensatory activation and a shift toward apoptosis. This pattern suggests that misfolded but partially functional missense proteins retain the capacity to initiate adaptive responses, whereas truncated products generated by PTC lack this ability, thereby enhancing pro-apoptotic signaling. *CHOP* upregulation has previously been linked not only to persistent ER stress but also to mitochondrial dysfunction [62]. The elevated *CHOP* levels observed in both p.Tyr109* and p.Cys114Trp imply that ER stress in these variants may be mechanistically associated with secondary mitochondrial impairment.

Subsequently, we assessed the subcellular localization of WISP3 by performing co-localization analysis using TOM20 as a mitochondrial marker. Previous studies have reported mitochondrial localization of WISP3 in human chondrocytes, and our findings are consistent with these observations [9]. The p.Cys52* variant exhibited markedly reduced WISP3 levels and low Pearson correlation coefficients, due to the absence of detectable WISP3 protein in the cells. Although the Manders’ M2 value appeared elevated, this likely reflected background signal rather than true spatial overlap.

The elevated mitochondrial superoxide levels observed in cells expressing p.Tyr109* and p.Cys114Trp suggest enhanced oxidative stress. In contrast to the wild-type variant, these variants showed increased mitochondrial localization, and such accumulation is predicted to impair mitochondrial proteostasis and interfere with the organelle’s protein-handling capacity. Mislocalized proteins within mitochondria are well established to promote ROS production, thereby reinforcing oxidative stress and activating the UPR [64]. This explanation links the observed ROS increase to altered subcellular targeting of WISP3 variants and is consistent with the ER stress marker expression detected in these cells.

These findings demonstrate that WISP3 variants disrupt redox balance, ER stress response, and subcellular localization in a variant-specific manner. The p.Tyr109* and p.Cys114Trp variants appear to impair ER–mitochondrial communication and compromise chondrocyte homeostasis through increased mitochondrial superoxide, UPR activation, and abnormal mitochondrial accumulation. In contrast, the p.Cys52* variant exhibited limited effects, likely reflecting loss of function due to the absence of protein. This integrative analysis underscores the role of WISP3 in maintaining intracellular stress balance and its contribution to the pathogenesis of PPD.

Transcriptional differences between wild-type or disease-associated WISP3 variants were evaluated through transcriptomic profiling. Compared with wild-type WISP3, cells expressing p.Cys52* or p.Cys114Trp displayed distinct gene expression changes. In both cases, the number of downregulated genes exceeded that of upregulated genes by 1.76- and 1.78-fold, respectively, indicating a general trend toward transcriptional suppression. Enrichment analysis revealed that these changes were largely variant-specific. While wild-type WISP3 expression promoted genes involved in cytoskeletal organization and ER-directed protein trafficking, these pathways were suppressed in cells expressing the variants. These findings suggest that the structural and trafficking functions of WISP3 are disrupted in a variant-dependent manner. According to the gene–pathway analysis (Figure 7E), many transcripts were linked to multiple biological categories, suggesting that the effects of the variant extend beyond specific pathways and impact broader regulatory networks.

The p.Cys114Trp variant induced widespread transcriptional activation of genes associated with ER stress, the UPR, and programmed cell death. Among the upregulated transcripts were key ER chaperones such as *HSPA5* and *DNAJB9*, which recognize misfolded proteins, along with *HERPUD1*, a component of the ER-associated degradation (ERAD) pathway [65,66,67]. The pro-apoptotic transcription factors *DDIT3 (CHOP)* and *ATF3* were also elevated under this condition [68,69]. Additionally, *PLCG2*, which is involved in calcium signaling and ROS production, may contribute to ER–mitochondrial stress responses, while *KLF4* is associated with adaptive stress signaling, cellular differentiation, and redox balance [70,71]. The coordinated induction of these transcripts supports the interpretation that p.Cys114Trp activates a stress response that encompasses both ER proteostasis and mitochondrial redox homeostasis.

In contrast, p.Cys52* resulted in a weaker transcriptional response without clear activation of canonical stress pathways, likely due to the absence of functional protein. Among the upregulated genes, only *CSNK1A1L*, *ANP32CP*, and *NME2P1* were prominently increased. *CSNK1A1L* has been linked to repression of Wnt signaling, *ANP32CP* is involved in apoptosis regulation and nucleocytoplasmic transport, and *NME2P1*, a predicted pseudogene, has been implicated in apoptotic processes [72,73,74]. These genes were elevated in both variant-expressing conditions but were not observed in cells expressing wild-type WISP3, suggesting a variant-specific transcriptional response.

### 3.4. Variant-Specific Disruption of WISP3 Interactome Reveals Divergent Stress Adaptation and Cytoskeletal Dysregulation Mechanisms in PPD

We performed proteomic mapping to identify intracellular interaction partners of WISP3 and to assess how disease-associated variants alter this interaction landscape. The full-length wild-type WISP3 protein was compared with the structurally destabilized missense variant p.Cys114Trp and the truncated isoform p.Tyr109*, which, despite harboring a PTC, is translationally expressed. The analysis revealed markedly different effects of the two WISP3 variants on the protein’s interaction profile. The p.Tyr109* variant, which yields a truncated isoform, led to a substantial reduction in the number of WISP3-associated proteins. This finding highlights the critical role of the C-terminal domains in mediating protein–protein interactions and maintaining the integrity of the WISP3 interactome. In contrast, despite its structural instability, the p.Cys114Trp variant exhibited a broader range of interactions compared with the wild-type protein. This increase may reflect a compensatory mechanism, possibly arising from conformational alterations or intracellular dysfunction. Together, these findings suggest that structural disruption reshapes WISP3’s molecular interactions and may underlie variant-specific pathological mechanisms.

In both transcriptomic and proteomic datasets, several cytoskeletal proteins—including ACTBM, TUBB1A, TUBB2A, TUBB2B, POTEF, and POTEI—were identified as interaction partners regardless of WISP3 variant. The beta-tubulin isoforms contribute to microtubule organization, while POTEF and POTEI are involved in ATP/protein/nucleotide binding and the regulation of cellular components [75]. ACTBM, a beta-actin isoform, supports cell shape, motility, and mechanical stability. Previous phylogenetic analysis places POTEF and POTEI in close evolutionary proximity to ACTBM, further supporting their functional similarity [75]. Downregulation of ACTBM at the transcript level in both variant-expressing cells suggests that variant-specific disruption of WISP3 may impair actin dynamics. The consistent detection of these proteins across all isoforms implies that WISP3 may operate through a conserved cytoskeletal module, which could contribute to chondrocyte dysfunction in PPD.

In contrast to these shared cytoskeletal interactions, LIMA1, which supports cell–cell adhesion and stabilizes the actin cytoskeleton through its interaction with adhesion complexes, was detected exclusively in the interactome of wild-type WISP3 [76,77]. This finding suggests that functional WISP3 may be required to maintain LIMA1 expression. Loss of LIMA1 disrupts the adhesion complexes, promotes cytoskeletal remodeling, and activates β-catenin signaling, potentially facilitating mesenchymal transitions. In the context of WISP3 dysfunction, reduced LIMA1 levels may further compromise cytoskeletal integrity and intercellular adhesion.

Calmodulin is a calcium-binding protein that plays critical roles in chondrogenesis and intracellular signaling [78,79]. In our proteomic analysis, CALM proteins were found only in cells expressing full-length WISP3 (wild-type and p.Cys114Trp) and were absent in truncation variants. This suggests that full-length WISP3 may preserve Ca^2+^–calmodulin interactions, whereas truncated forms fail to support this interaction. Given that WISP3-deficient cells exhibit elevated mitochondrial Ca^2+^ levels, the loss of interaction of calmodulin with the truncation variant may reflect a loss of buffering capacity, thereby exacerbating mitochondrial stress [9,80]. Consequently, impaired calmodulin signaling in these contexts may disrupt processes such as chondrocyte differentiation and matrix homeostasis, further contributing to cartilage dysfunction. These observations imply that WISP3 may function not only as a structural regulator but also as a modulator of intracellular calcium signaling and mitochondrial equilibrium.

Among the proteins identified, CKAP4 stood out as a notable interactor of WISP3. Previous studies have shown that CKAP4 interacts with DKK1 to form the DKK1–CKAP4–LRP5/6 complex, which suppresses Wnt signaling while simultaneously activating the PI3K/AKT pathway [81]. Similarly, WISP3 is known to bind LRP6 and Frizzled receptors, thereby negatively regulating both Wnt and BMP signaling pathways [47]. In our study, we identified a previously unrecognized interaction between full-length WISP3 (both wild-type and p.Cys114Trp) and CKAP4. This finding suggests that WISP3 may integrate into the DKK1–CKAP4–LRP6–Frizzled complex and participate in a broader multi-receptor network that modulates Wnt, BMP, and PI3K/AKT signaling axes.

Beyond its role in intracellular signal transduction, CKAP4 is also involved in maintaining ER homeostasis [82]. In cells expressing the p.Cys114Trp variant, we observed increased CKAP4 interaction. While this elevation may reflect an adaptive response to restore ER proteostasis, structural disruptions caused by the variant could limit its effectiveness, thereby triggering mitochondrial dysfunction [83]. The preserved interaction between CKAP4 and the p.Cys114Trp variant, despite increased interaction levels, indicates that WISP3 may influence multiple signaling networks and that variant-dependent perturbations could compromise their integrity. These observations underscore the need for further investigation into the structural and functional properties of the proposed WISP3–CKAP4–DKK1–LRP6–Frizzled complex under both physiological and pathological conditions.

Detection of DDX5 and DDX17, specifically in association with the p.Cys114Trp variant, suggests that this WISP3 isoform may engage in novel and potentially pathogenic intracellular interactions. DDX5 expression has been associated with the regulation of ER stress and autophagy, while both DDX5 and DDX17 are known to modulate Wnt/β-catenin signaling pathways [84,85]. Their exclusive interaction with the p.Cys114Trp variant is consistent with the elevated cellular stress observed in this context and may also reflect structural destabilization of the variant protein, facilitating aberrant binding events.

Lastly, HSPB1 functions as an ATP-independent chaperone that binds misfolded proteins, preventing aggregation and proteotoxic stress [86]. It also mitigates oxidative damage, stabilizes the actin cytoskeleton, and neutralizes mitochondrial death signals [87]. These cytoprotective roles are essential in cartilage, where declining HSPB1 levels accelerate chondrocyte apoptosis during osteoarthritis [88]. Its selective association with p.Cys114Trp suggests a compensatory response to the proteostatic burden imposed by this variant, which may promote chondrocyte loss and joint degeneration in PPD.

Our findings suggest that the absence of WISP3 or the presence of structurally unstable variants can disrupt chondrocyte function through distinct mechanisms, thereby contributing to the pathogenesis of PPD. While the type of disruption varies, complete loss of the protein versus production of misfolded or unstable forms triggering different cellular responses, these heterogeneous mechanisms appear to converge on shared downstream consequences. Rather than acting through a single signaling axis, WISP3 appears to function as a multifaceted regulator involved in maintaining intracellular homeostasis, skeletal organization, and cytoskeletal integrity. This is consistent with its extensive interactome network and its previously suggested role as a ligand transmitting autocrine or paracrine signals, which may provide a unifying explanation for how diverse molecular perturbations can give rise to overlapping clinical phenotypes. PPD is a rare disease that is often diagnosed late in childhood; therefore, variant-specific molecular differences may exist at earlier stages, but as the disease progresses, disruption of cartilage structure may become the dominant factor, potentially leading to overlapping clinical outcomes. The observed alterations point toward dysregulation of the Wnt/β-catenin pathway as a central contributor, and our results offer new insights into the complexity of this regulatory network. Therefore, understanding the effects of specific variants, as highlighted in our study, may help inform the development of alternative therapeutic strategies when the disease is diagnosed at earlier stages.

This study has several limitations. The number of biological replicates in RNA-seq and LC-MS/MS analyses was limited due to budgetary constraints, reducing statistical power. In addition, reliance on an overexpression model may not fully reflect physiological regulation or variant-specific effects. Future studies with larger sample sizes and CRISPR/Cas9-edited cell lines, the development of organoid models, and in vivo validation will be essential to strengthen these findings and clarify the pathogenic consequences of WISP3 dysfunction. While patient-derived cells would provide valuable translational insight, obtaining primary chondrocytes is technically challenging and not a routine procedure, which limits their accessibility for research purposes. Nevertheless, the integration of structural predictions, functional assays, and omics profiling provides a broad and coherent experimental framework that captures distinct aspects of WISP3 dysfunction. The combination of these complementary approaches enables a more complete view of how different variants alter cellular stress responses, redox balance, and extracellular matrix regulation. In doing so, this work offers a depth of mechanistic understanding that is rarely achieved in rare skeletal disorders and establishes a foundation that can inform both future research and potential translational applications. Nevertheless, the integration of structural predictions, functional assays, and omics profiling provides a broad and coherent experimental framework that captures distinct aspects of WISP3 dysfunction. The combination of these complementary approaches enables a more complete view of how different variants alter cellular stress responses, redox balance, and extracellular matrix regulation. In doing so, this work offers a depth of understanding that is rarely achieved in rare skeletal disorders and establishes a foundation that can inform both future research and potential translational applications. Further studies are warranted to fully elucidate the molecular landscape shaped by WISP3 dysfunction in skeletal disease.

## 4. Materials and Methods

### 4.1. Cell Culture Experiments

Human chondrocytes (Cat. No. 402-05A, Cell Applications, Inc., San Diego, CA, USA) were cultured in high-glucose Dulbecco’s Modified Eagle Medium (DMEM) (Cat. No. 11965092, Gibco, Waltham, MA, USA) supplemented with 10% fetal bovine serum (FBS) (Cat. No. 10270106, Gibco, Waltham, MA, USA) and 1% penicillin–streptomycin (Cat. No. 15140122, Gibco, Waltham, MA, USA) at 37 °C in a humidified incubator with 5% CO_2_. Authentication was confirmed by Alcian Blue staining and morphological assessment. Cells were used up to passage 16, as higher passages showed morphological changes suggestive of dedifferentiation. For transfection studies, 2.5 × 10^5^ cells were seeded in 12-well plates, and the following day, cells were transfected with 1 µg of plasmid DNA using LipofectMax (Cat. No. FP318, ABP Biosciences, Rockville, MD, USA) in 500 µL of 10% FBS-DMEM, following the manufacturer’s instructions. After 4 h, the transfection volume was brought up to 1 mL by adding fresh medium to minimize cytotoxicity and support post-transfection recovery [44].

### 4.2. Protein Structure and Prediction of Protein Stability

Using the only AlphaFold predicted structure of WISP3 (AF-O95389-F1-v4) first, it was investigated in terms of domain architecture to decide high pLDDT-containing, high-confidence regions that were well matched with our variants utilized in this study. It was apparent that related variants were clustered in the IGFBP N-terminal domain of WISP3; hence, this domain was isolated from this structure. After isolation, FoldX program (version 5.0) was used to repair this structure in terms of bad torsion angles, Van der Waals clashes, or total energy (repairPDB command) [23]. The repaired structure was fed into theBuildModel command of FoldX for prediction of variants’ impact on protein stability. ΔΔG of protein stability (kcal/mol) values that were higher than +0.5 kcal/mol were considered as highly destabilizing mutations. Visualization and modification of the WISP3 structure were handled through VMD software (version 1.9.4) [89].

### 4.3. Variant Construct Generation

Site-directed mutagenesis was employed to generate *CCN6* variant constructs using Q5 High-Fidelity DNA Polymerase (Cat. No. M0491, New England Biolabs, Hertfordshire, UK), in accordance with the manufacturer’s instructions. The plasmid template containing the full-length human *WISP3* cDNA ORF clone (Cat. No. HG15729-NY) was obtained from Sino Biological (Beijing, China). Variant-specific primers were designed using PrimerX (http://www.bioinformatics.org/primerx accessed on 20 July 2025) and synthesized with 5′ phosphorylation by Oligomer (Çankaya, Türkiye). Details on primer sequences, GC content, and optimal annealing temperatures are provided in Table 2.

Following amplification, polymerase chain reaction (PCR) products were incubated with *DpnI* endonuclease (Cat. No. R0176, New England Biolabs, Hertfordshire, UK) at 37 °C for 2 h to selectively digest the methylated parental plasmid. The digested reaction mixtures were then transformed into the chemically competent *E. coli* DH5α strain by heat-shock. Transformed cells were plated on LB agar supplemented with kanamycin (Cat. No. K-120-5, GoldBio, St Louis, MO, USA) for selection. Sanger sequencing (Macrogen, Europe, Amsterdam, Netherlands) using T7 promoter, forward primer (5′–TAATACGACTCACTATAGGG–3′), and BGH terminator (5′–TAGAAGGCACAGTCGAGG–3′) reverse primers was performed to confirm the presence of the variations.

### 4.4. MTT Assay

Human chondrocytes (5 × 10^4^ cells/well) were seeded into 96-well plates one day prior to transfection. Forty-eight hours after transfection, wells were gently washed with Dulbecco’s Phosphate-Buffered Saline (DPBS) (Cat. No. 14190144, Gibco, Waltham, MA, USA), and MTT reagent (Cat. No. M6494, Invitrogen, Waltham, MA, USA) was freshly prepared in PBS at a stock concentration of 5 mg/mL. The reagent was then added to each well in phenol red-free DMEM (Cat. No. 31053028, Gibco, Waltham, MA, USA) supplemented with 10% FBS to achieve a final concentration of 0.5 mg/mL. After a 4 h incubation at 37 °C, the medium was removed, and 100 µL of dimethyl sulfoxide (DMSO) (Cat. No. 116743, Sigma-Aldrich, St. Louis, MO, USA) was added to each well to dissolve the purple formazan crystals. Plates were incubated on a shaker at room temperature in the dark for 15 min. Absorbance was measured at 570 nm using a SPECTROstar Nano microplate reader (BMG Labtech, Ortenberg, Germany). Each condition was tested in eight biological replicates, measured in duplicate. Raw absorbance values were used for comparative analysis across experimental groups.

### 4.5. Adhesion Assay

96-well plates were coated with 50 μg/mL human type I collagen (Cat. No. 07005, Stemcell Technologies, Vancouver, BC, Canada) for 1 h at room temperature. Wells were washed twice with phosphate-buffered saline (PBS) and blocked with 1% bovine serum albumin (BSA) (Cat. No. BSA-1S, Capricorn Scientific, Ebsdorfergrund, Germany) for 60 min at 37 °C. After a final PBS wash, 10 × 10^4^ cells were seeded per well in 50 µL serum-free, phenol red-free DMEM and incubated at 37 °C for 30 min. Non-adherent cells were removed by gentle PBS washing. Adherent cells were fixed with 4% paraformaldehyde (PFA) for 15 min, stained with crystal violet (Cat. No. C0775, Sigma Aldrich, St. Louis, MO, USA) for 20 min, and rinsed thoroughly with tap water. After air drying, crystal violet was solubilized in 2% sodium dodecyl sulfate (SDS) for 30 min at room temperature. Absorbance was measured at 550 nm using a SPECTROstar Nano microplate reader (BMG Labtech, Ortenberg, Germany) [90]. The assay was performed using three biological replicates.

### 4.6. Migration and Wound Healing Assay

24 h after transfection, 1 × 10^5^ cells were seeded into each well of a 24-well plate and incubated overnight. On the following day, a linear scratch was introduced across the cell monolayer using a sterile 1000 μL pipette tip. Detached cells were removed by washing the wells with DPBS, and fresh 10% FBS-DMEM was added. Phase-contrast images were captured at defined time points using a Zeiss Axiocam (Carl Zeiss, Oberkochen, Germany) microscope system. Wound healing efficiency was quantified by analyzing scratch closure using ImageJ software (v1.54p, NIH, Bethesda, MD, USA), based on the percentage reduction in wound area at 24 h relative to the initial wound width, calculated as follows: 100 − (((initial wound area − 24 h wound area)/initial wound area) × 100). The assay was performed using three biological replicates.

### 4.7. RNA Isolation and Quantitative PCR

Total RNA was obtained from cells using TRIzol Reagent (Cat. No. 15596026, Thermo Fisher Scientific, Waltham, MA, USA), following the manufacturer’s guidelines. For complementary DNA (cDNA) synthesis, 500 ng of total RNA was reverse-transcribed using the SensiFAST cDNA Synthesis Kit (Cat. No. BIO-65053, Cincinnati, OH, USA) according to the manufacturer’s instructions. qPCR was performed on a LightCycler 96 System (Roche, Mannheim, Germany) using the SensiFAST SYBR No-ROX Kit (Cat. No. BIO-98005, Meridian Bioscience, Cincinnati, OH, USA). The reaction was carried out in a 10 µL volume with 50 ng of cDNA and 0.4 µM of each primer. The amplification protocol included an initial denaturation at 95 °C for 120 s, followed by 40 cycles of 95 °C for 5 s, 60 °C for 10 s, and 72 °C for 15 s. At the end of the analysis, melting curve analysis was included to confirm the specificity of the amplifications. Relative gene expression levels were calculated using the 2^−ΔΔCt^ method, with β-actin *(ACTB)* used as the internal reference gene [91]. The primers used for qPCR are listed in Table 3. The assay was performed using three biological replicates.

### 4.8. Gelatin Zymography

Gelatinase assessment was performed using 12.5% SDS-PAGE gels containing 1 mg/mL gelatin (Cat. No. G2500, Sigma-Aldrich, Waltham, MA, USA). Post-transfection, 10 μg of serum-free supernatants were mixed with 5× non-reducing loading dye (0.01% bromophenol blue, 4% SDS, 125 mM Tris-HCl (pH 6.8), 20% glycerol) and electrophoresed at 4 °C until the dye front reached the bottom of the gel. Following electrophoresis, gels were washed in a solution of 2.5% Triton X-100 in 50 mM Tris–HCl (pH 7.5), 5 µM CaCl_2_, and 1 µM ZnCl_2_ for 1 h to remove SDS and enable enzyme renaturation, followed by a brief rinse with double-distilled water. Gels were then incubated overnight at 37 °C in incubation buffer containing 5 mM CaCl_2_, 1 µM ZnCl_2_, 1% Triton X-100, and 50 mM Tris–HCl (pH 7.5) [92]. The next day, gels were stained with Coomassie Brilliant Blue G-250 (Cat. No. CBB555, BioShop Canada, Burlington, ON, Canada) for 1 h and destained using a solution of 40% methanol and 10% glacial acetic acid until clear lytic bands were visible. Band intensity levels were analyzed using densitometric plots via ImageJ software (version: v1.54p, NIH, USA). The assay was performed using six biological replicates.

### 4.9. Flow Cytometry

Forty-eight hours from transfection, cells were co-stained with MitoTracker Green FM (Cat. No. 9074, Cell Signaling Technologies, Danvers, MA, USA) at a final concentration of 250 nM and MitoSOX Red mitochondrial superoxide indicator (Cat. No. C259, ABP Biosciences, Rockville, MD, USA) at 2.5 µM. Staining was performed in serum-free DMEM at 37 °C for 20 min in the dark. Then, cells were washed with PBS and collected using 0.25% trypsin-EDTA, neutralized with DPBS, and centrifuged at 300× *g* for 5 min. Pelleted cells were rinsed with PBS and Hanks’ Balanced Salt Solution (HBSS) (Cat. No. HBSS-1A, Capricorn Scientific, Ebsdorfergrund, Germany). A total of 20,000 events per sample were recorded using a BD FACSCanto II flow cytometer. Fluorescence was detected in the FITC and PE channels for MitoTracker Green and MitoSOX Red, respectively. Compensation was applied based on single-stained controls. Data analysis was performed using FlowJo software (v10.10.0). The assay was performed using two biological replicates.

### 4.10. Confocal Microscopy

For immunofluorescence imaging, cells were seeded on 0.1% gelatin-coated coverslips one day after transfection and allowed to adhere overnight. The following day, cells were fixed in 4% paraformaldehyde (PFA) and permeabilized with 0.1% Triton X-100 in PBS. Blocking was performed using 5% normal goat serum (Cat. No. G-1B, Capricorn Scientific, Ebsdorfergrund, Germany). Cells were incubated overnight at 4 °C with 1:1200 anti-HA (Cat. No. 66006-2-Ig, Proteintech, Rosemont, IL, USA) and 1:400 anti-TOM20 (Cat. No. 11802-1-AP, Proteintech, Rosemont, IL, USA). After PBST washes, 1:1000 Alexa Fluor 488-conjugated (Cat. No. 4412, Cell Signaling Technology, Danvers, MA, USA) and 1:400 CoraLite 594-conjugated (Cat. No. SA00013-3, Proteintech, Rosemont, IL, USA) were used as secondary antibodies. Nuclei were counterstained with 300 nm DAPI (Cat. No. 14285, Cayman Chemical, Ann Arbor, MI, USA). Confocal images were acquired using a Zeiss LSM 700 microscope. Z-projected stacks (maximum intensity) were analyzed in ImageJ (version v1.54p, NIH, USA) using the BIOP JaCoP plugin (version v1.2.0) [93]. All images were acquired using identical microscope settings. Co-localization between HA-tagged WISP3 and TOM20 was quantified by calculating Pearson correlation and Manders’ M2 coefficients. Prior to analysis, each channel was thresholded using the Otsu method, a histogram-based algorithm that does not account for inter-channel correlation. Median values were obtained from ≥25 cells per replicate across three independent experiments.

### 4.11. Western Blot

Total cellular proteins were isolated using radioimmunoprecipitation assay buffer (RIPA) (Cat. No. 89901, Thermo Fisher Scientific, Waltham, MA, USA) supplemented with protease inhibitors (Cat. No. AR1182, Boster Bio, Pleasanton, CA, USA) and protein concentration was quantified using the Pierce BCA Protein Assay Kit (Cat. No. 23227, Thermo Fisher Scientific, Waltham, MA, USA), according to the manufacturer’s instructions. 20 µg of protein samples were separated by 4–12–15% SDS-PAGE and transferred onto 0.22 µm polyvinylidene difluoride (PVDF) membranes (Cat. No. 88520, Thermo Fisher Scientific, Waltham, MA, USA). Membranes were blocked for 1 h at room temperature in 0.05% TBS-T containing 5% BSA (Cat. No. BSA-1S, Capricorn Scientific, Ebsdorfergrund, Germany). Primary antibody incubation was performed overnight at 4 °C using antibodies diluted in blocking buffer: 1:4000 anti-HA (Cat. No. 51064-2-AP, Proteintech, Rosemont, IL, USA) and 1:2000 β-Tubulin (Cat. No. A12289, Abclonal, Woburn, MA, USA). The next day, membranes were incubated for 1 h at room temperature with 1:5000 horseradish peroxidase (HRP)-conjugated anti-rabbit IgG(H+L) secondary antibody (Cat. No. AS014, Abclonal, Woburn, MA, USA). Membranes were visualized using a chemiluminescence (ECL) substrate kit (Cat. No. 1705060, Bio-Rad, Hercules, CA, USA) and imaged using the ChemiDoc Imaging System with Image Lab software (Bio-Rad, Hercules, CA, USA). The assay was performed using three biological replicates.

### 4.12. RNA-Seq Quantification, Differential Gene Expression, and Enrichment Analysis

Total RNA was extracted from *CCN6* overexpressed cells *(OE)* and from two pathogenic *CCN6* variants p.Cys52* and p.Cys114Trp, using Qiagen RNeasy kit (Cat. No. 74104, Qiagen, Hilden, Germany), each represented by two independent biological replicates. Ribosomal-depleted next-generation sequencing libraries were prepared using Illumina Stranded Total RNA Prep, Ligation with Ribo-Zero Plus kit (Cat. No. 20040529, Illumina, San Diego, CA, USA). The libraries were sequenced on the Illumina NovaSeq 6000 platform (2 × 100 cycles) with a total of 25 M fragment reads. The raw reads were quality-filtered before alignment to the GRCh38 reference genome with HISAT2 (v2.2.1) (default splice-aware settings) using GENCODE v48 comprehensive annotations [94,95]. Gene-level counts were quantified with featureCounts (2.0.8), after which a variance-stabilized expression matrix was subjected to principal component analysis and pairwise Pearson correlation, using the stats package (v4.2.2) in R.

Genes were retained only when they reached ≥1 CPM (Counts per million) (≈10 raw reads) in at least the two samples comprising the smallest genotype group and were detectable at this threshold in ≥70% of samples within every group. After which gene-level counts were analyzed with edgeR’s quasi-likelihood pipeline (v4.0.16), tag-wise dispersions were estimated while accounting for biological replicate structure, differential expression was assessed by contrasting each variant genotype separately against the wild-type reference, and adjusted *p*-values were computed using the default Benjamini–Hochberg method for false discovery rate control [96]. Significant genes (FDR < 0.05) were ranked and analyzed with clusterProfiler’s fgsea implementation (v4.6.2) against the MSigDB (v2025.1.Hs) GO gene sets to uncover enriched biological processes [97,98]. Z-score-scaled expression of ER response-related, up- and down-regulated genes per contrast was visualized with pheatmap (v1.0.12), providing an intuitive heatmap overview of expression trends across all six samples [99]. Volcano plots were generated using the ggplot2 package (v3.5.2), while upset plots and dot plots were created using the clusterProfiler [100].

### 4.13. Co-Immunoprecipitation and LC–MS/MS Analysis

Human chondrocytes were transfected with plasmids encoding HA-tagged wild-type WISP3 (OE), p.Tyr109, or p.Cys114Trp variants, each represented by two independent biological replicates. After transfection, co-immunoprecipitation was performed using the Pierce Direct Magnetic IP/Co-IP Kit (Cat. No. 88828, Thermo Fisher Scientific, Waltham, MA, USA), following the manufacturer’s instructions. For immunoprecipitation, 1 mg of total protein lysate was incubated with 5 μg of mouse monoclonal anti-HA antibody (Cat. No. 66006-2-Ig, Proteintech, Rosemont, IL, USA) covalently coupled to magnetic beads via NHS-activated chemistry. Binding was performed at room temperature under gentle rotation. After extensive washing to remove non-specific proteins, IP complexes were eluted using the provided elution buffer supplemented with 10% neutralization buffer.

Protein extraction was performed using the UPX Protein Extraction Kit (Cat. No. ab270054, Abcam, Waltham, MA, USA). Samples were diluted and homogenized according to the manufacturer’s instructions. Denaturation was achieved by heating the samples at 95 °C for 5 min. The denatured samples were then transferred to 30 kDa molecular weight cut-off FASP columns (Cat. No. MRCFOR30, Merck, Darmstadt, Germany). Alkylation was performed by adding 400 mM iodoacetamide to the columns, followed by incubation in the dark at room temperature for 20 min. After alkylation, the columns were washed three times with 8 M urea and subsequently three times with 50 mM ammonium bicarbonate to remove any residual reagents and urea. Protein digestion was carried out by adding 1 µg of trypsin (Cat. No. 90057, Thermo Scientific, Waltham, MA, USA) to each sample, followed by overnight incubation at 37 °C. After digestion, peptides were recovered by sequential elution: two washes with 50 mM ammonium bicarbonate and one wash with 500 mM sodium chloride. The resulting peptide-containing eluates were collected, and peptide concentrations were quantified using the Pierce Quantitative Colorimetric Peptide Assay (Cat. No. 23275, Thermo Scientific, Waltham, MA, USA) following the manufacturer’s protocol.

NanoLC–MS/MS analysis was conducted using an ACQUITY UPLC M-Class system coupled to a Xevo G2-XS Q-Tof mass spectrometer (Waters, Milford, MA, USA). Peptide mixtures were initially loaded onto a trap column (Symmetry C18, 5 µm, 180 µm i.d. × 20 mm) at a flow rate of 3 µL/min for 2 min using 5% mobile phase B (acetonitrile containing 0.1% formic acid). Mobile phase A consisted of water with 0.1% formic acid. The separation of peptides was then performed on a CSH C18 column (1.7 µm, 75 µm i.d. × 250 mm), maintained at 45 °C with a flow rate of 0.3 µL/min. Chromatographic separation was achieved with a 120 min run time, including a 90 min linear gradient from 5% to 45% acetonitrile. Eluted peptides were detected by UV absorbance at 214 nm prior to mass spectrometric analysis. The mass spectrometer was equipped with an electrospray ionization (ESI) source operating in positive ion mode, employing a data-independent acquisition (DIA) method in sensitivity mode. The scan range was *m*/*z* 50–2000 with a scan time of 0.5 s. A ramped collision energy of 14–40 V was applied for high-energy scans. [Glu1]-Fibrinopeptide B (*m*/*z* 785.8426) was continuously infused during data acquisition to serve as a lock mass for correction of mass accuracy drift.

Data were processed using Progenesis QI for Proteomics software (v4.1) with the reviewed UniProt database (release date: 18 June 2025, UniProtKB 2025_03). Peak intensity thresholds were set to 150 counts for low-energy and 50 counts for high-energy conditions. Search parameters required a minimum of 3 fragment ion matches per peptide, 7 fragment ion matches per protein, and at least 1 unique peptide per protein. The maximum number of allowed missed cleavages for tryptic digestion was 1. Carbamidomethylation of cysteine was specified as a fixed modification, while oxidation of methionine and deamidation of asparagine/glutamine were set as variable modifications.

Label-free quantitative proteomics data from two biological replicates per condition (p.Tyr109* or p.Cys114Trp vs. WT) were analyzed using the DEP v1.28.0 package in R, which incorporates the limma framework for differential expression analysis. Protein intensity data were cleaned, log_2_-transformed after pseudocount addition, and normalized by mean centering across samples. Proteins missing entirely in any one condition were excluded, and remaining missing values were imputed using a left-censored probabilistic approach (MinProb), assuming values were below detection thresholds. Differential expression testing was performed using linear models with empirical Bayes moderation, treating WT as the reference condition. No fold-change threshold was applied during significance testing to preserve sensitivity. Volcano plots were generated to visualize log_2_ fold changes against statistical significance via ggplot2 [101].

Protein interaction networks for wild-type WISP3 (OE) and two variants (p.Tyr109* and p.Cys114Trp) were constructed using interactor lists derived from co-immunoprecipitation mass spectrometry. Interactors unique to each condition and shared across combinations were compiled. A graph-based network was built using tidygraph v1.3.1 and visualized with ggraph v2.2.1 in R [102,103]. Variant nodes were connected to their respective interactors, and edge colors indicated the pattern of condition overlap. Protein nodes were annotated with functional categories and displayed as pie charts using ggforce v0.5.0, allowing multi-category visualization [103]. Node sizes reflected their connectivity, and layout was computed using the Fruchterman–Reingold algorithm. Protein labels were repelled to reduce overlap, and variant nodes were displayed as labeled tiles [104].

### 4.14. Statistical Analysis

Statistical analyses were executed in Python 3.12 (NumPy 1.26, SciPy 1.13) [105]. For each experimental group, pcDNA3.1 control, variants, and overexpression, outliers were removed using the interquartile-range criterion (values falling outside Q1 or Q3 ± 1.5 × IQR). Welch-corrected one-way ANOVA was reported alongside the standard fixed-effects ANOVA (α = 0.05). Significant omnibus results were followed by Tukey’s honestly significant difference procedure to obtain multiplicity-adjusted pairwise comparisons (95% confidence). Relative transcript abundance was quantified with the 2^−ΔΔCt^ method. Quantification-cycle (Cq) readings from the duplicate technical reactions were first averaged and then used to compute the intra-sample normalization factor, ΔCt, defined as ΔCt = Cq_target − Cq_*ACTB*. Inter-condition normalization was subsequently achieved by calculating ΔΔCt = ΔCt_sample − ΔCt_calibrator, where ΔCt_calibrator represents the mean ΔCt of the control samples [91]. qPCR data are presented as fold-change values with 95% confidence intervals (CI). One-way ANOVA (α = 0.05) was performed on fold changes, and group differences were resolved with a Tukey honestly significant difference post hoc test to identify significant pairwise contrasts among the groups Box-and-whisker and bar plots with jittered raw points were produced in Matplotlib v3.9/Seaborn v0.14 [106], and pairwise differences were annotated with asterisks: * *p* < 0.05, ** *p* < 0.01, *** *p* < 0.001, **** *p* < 0.0001, and “ns” for *p* ≥ 0.05.

## 5. Conclusions

This study presents the first functional characterization of four *CCN6* variants—p.Cys52*, p.Tyr109*, p.Gly83Glu, and p.Cys114Trp—linked to PPD. Using an integrative approach combining cellular phenotyping, transcriptomics, and proteomics, we uncovered distinct variant-specific effects on chondrocyte function. Although all variants map to the IGFBP domain of WISP3, their molecular outcomes varied significantly. The p.Cys52* and p.Tyr109* variants led to absent or truncated proteins with reduced interaction capacity, consistent with loss-of-function mechanisms. In contrast, p.Cys114Trp produced a structurally unstable full-length protein associated with aberrant interactions, stress activation, and widespread transcriptomic changes, indicating a gain-of-function effect. p.Gly83Glu, previously regarded as benign, showed minimal phenotypic and regulatory disruption. These findings reveal that WISP3 dysfunction in PPD involves diverse mechanisms—including both loss and gain of function—highlighting the need for functional validation in interpreting uncertain variants in skeletal diseases.

## Figures and Tables

**Table 1:** FoldX-based ΔΔG estimation (protein stability) of WISP3 IGFBP N-terminal domain variants. Positive values indicate decreased protein stability.

Variant	ΔΔG Prediction for Protein Stability (kcal/mol)
p.Gly83Glu	+2.73
p.Cys114Trp	+43.33

**Table 2:** Primer Sequences and GC Content Used for Site-Directed Mutagenesis.

Nucleotide Change	Protein Change	Forward and Reverse Primer (5′ → 3′)	GC Content (%)
c.156C>A	p.Cys52*	Fw: GTTTTGTCACTGGCCCTGAAAATGCCCTCAGCAGAAG	51
		Rv: CTTCTGCTGAGGGCATTTTCAGGGCCAGTGACAAAAC	
c.248G>A	p.Glu83Glu	Fw: CTGTGCCAAGCAACCAGAGGAAATCTGCAATGAAG	49
		Rv: CTTCATTGCAGATTTCCTCTGGTTGCTTGGCACAG	
c.327C>A	p.Tyr109*	Fw: GACAGGCCTAGGTAAGAGACTGGAGTGTG	55
		Rv: CACACTCCAGTCTCTTACCTAGGCCTGTC	
c.342T>G	p.Cys114Trp	Fw: GAGACTGGAGTGTGGGCATACCTTGTAGC	55
		Rv: GCTACAAGGTATGCCCACACTCCAGTCTC	

**Table 3:** Genes and primer sequences used for RT-PCR analysis.

Gene	Forward and Reverse Primer (5′ → 3′)
*CCN6*	Fw: ACTGTAGCCTGGAACCATTACT Rv: TGGTCACCCTGTTAGATATTCCC
*CHOP*	Fw: GGAAACAGAGTGGTCATTCCC Rv: CTGCTTGAGCCGTTCATTCTC
*sXBP1*	Fw: GCTGAGTCCGCAGCAGGT Rw: CTGGGTCCAAGTTGTCCAGAAT
*uXBP1*	Fw: CAGACTACGTGCACCTCTGC Rv: CTGGGTCCAAGTTGTCCAGAAT
*ACTB*	Fw: CACCATTGGCAATGAGCGGTTC Rv: AGGTCTTTGCGGATGTCCACGT

## Data Availability

Raw and processed RNA sequencing data have been deposited in the NCBI Sequence Read Archive (SRA; BioProject: PRJNA1300445) and Gene Expression Omnibus (GEO; accession: GSE304437) databases.

## Supplementary Materials

The following supporting information can be downloaded at https://www.mdpi.com/article/10.3390/ijms26188838/s1.

## Abbreviations

The following abbreviations are used in this manuscript:

PPD	Progressive pseudorheumatoid dysplasia
OMIM	Online Mendelian Inheritance in Man
*CCN6*	Cellular Communication Network Factor 6
WISP3	Wnt1-inducible-signaling pathway protein 3
JIA	Juvenile idiopathic arthritis
ESR	Erythrocyte Sedimentation Rate
CRP	C-Reactive Protein
ECM	Extracellular Matrix
IGFBP	Insulin-like Growth Factor Binding Protein
TSP1	Thrombospondin Type 1 Repeat
CTCK	Cysteine Knot-Containing Domain
Wnt	Wingless/Integrated
TGF-β	Transforming Growth Factor Beta
CHOP	C/EBP Homologous Protein
sXBP1	Spliced X-box Binding Protein 1
uXBP1	Unspliced X-box Binding Protein 1
MMP	Matrix Metalloproteinase
