# Supplementary material for: Molecular Consequences of CCN6 Variants Encoding WISP3 in Progressive Pseudorheumatoid Dysplasia
Source: Int J Mol Sci. 2025 Sep 11;26(18):8838. doi: 10.3390/ijms26188838 (PMC12469775; doi:10.3390/ijms26188838)
Supplement: Supplementary file 1 [file ijms-26-08838-s001.zip › ijms-3831684-supplementary.pdf]

Supplementary Figure S1

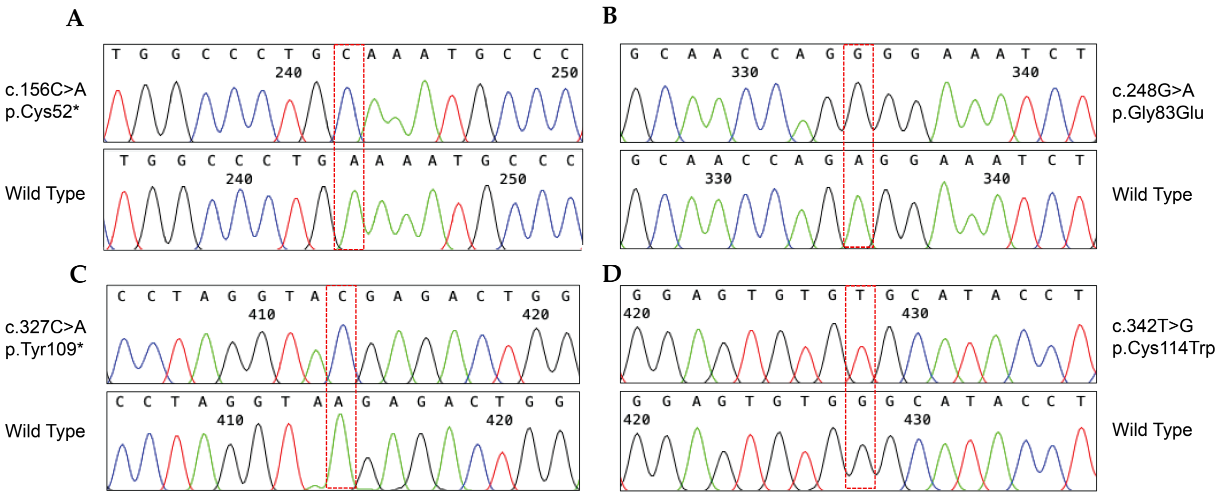

Supplementary Figure S1. Sanger sequencing electropherograms confirming the presence of *CCN6* single nucleotide variants introduced by site-directed mutagenesis. Each panel displays the corresponding nucleotide substitution compared to the wild-type sequence: **(A)** c.156C>A (p.Cys52*), **(B)** c.248G>A (p.Gly83Glu), **(C)** c.327C>A (p.Tyr109*), and **(D)** c.342T>G (p.Cys114Trp). Arrows indicate the location of the nucleotide change. Wild-type chromatograms are shown below each variant for comparison.

Supplementary Figure S2

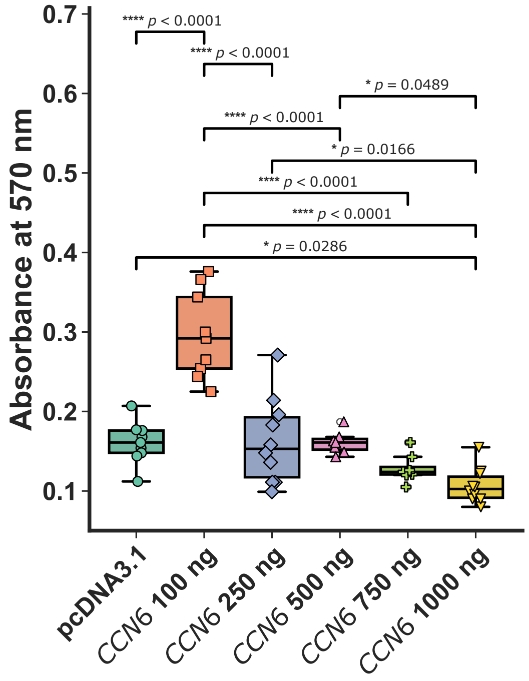

Supplementary Figure S2. Effect of increasing amounts of *CCN6* overexpression on cell viability in human chondrocytes. Boxplot showing MTT assay results following transfection of 402-05A cells with increasing amounts of wild-type *CCN6* plasmid (100–1000 ng). Cell viability was assessed 48 hours post-transfection by measuring absorbance at 570 nm. A dose-dependent decrease in cell viability was observed with increasing *CCN6* expression, with the highest viability detected in cells transfected with 100 ng plasmid. Statistical comparisons were performed using one-way ANOVA with Tukey's post hoc test. Significance is represented as *$p < 0.05$, ** $p < 0.01$, *** $p < 0.001$, **** $p < 0.0001$.

Supplementary Figure S3

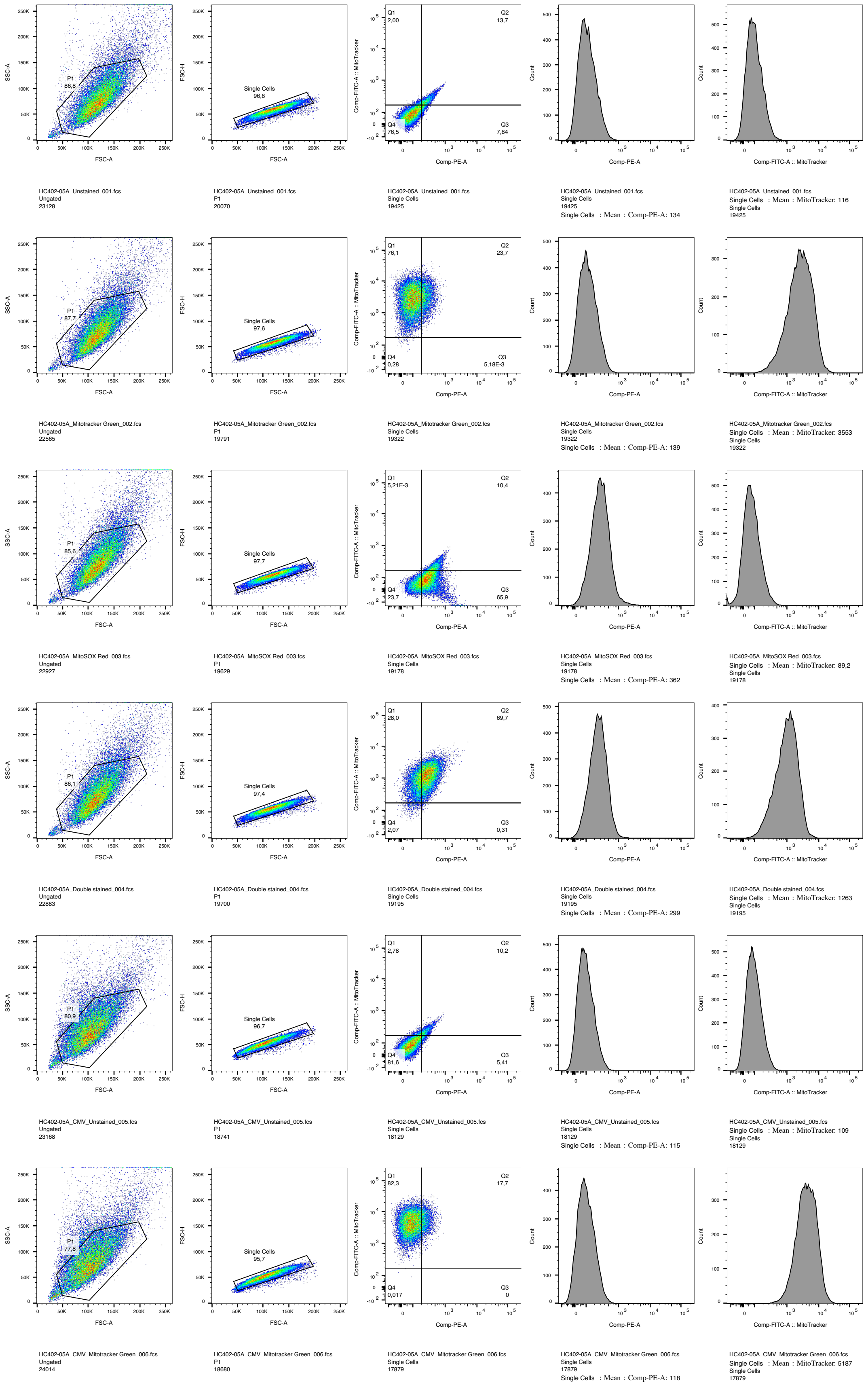

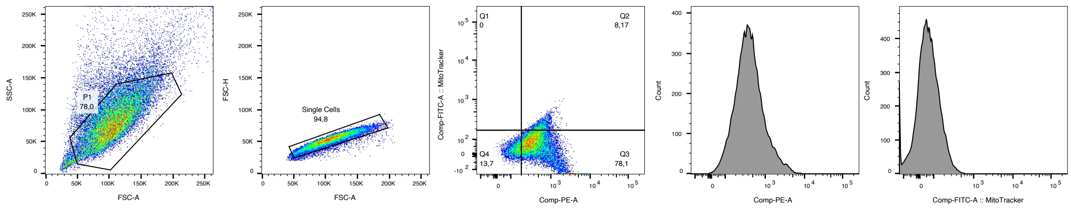

HC402-05A_CMV_MitoSOX Red_007.fcs
Ungated
24500

HC402-05A_CMV_MitoSOX Red_007.fcs
P1
19098

HC402-05A_CMV_MitoSOX Red_007.fcs
Single Cells
18113

HC402-05A_CMV_MitoSOX Red_007.fcs
Single Cells
18113
Single Cells : Mean : Comp-PE-A: 599

HC402-05A_CMV_MitoSOX Red_007.fcs
Single Cells : Mean : MitoTracker: 71,2
Single Cells
18113

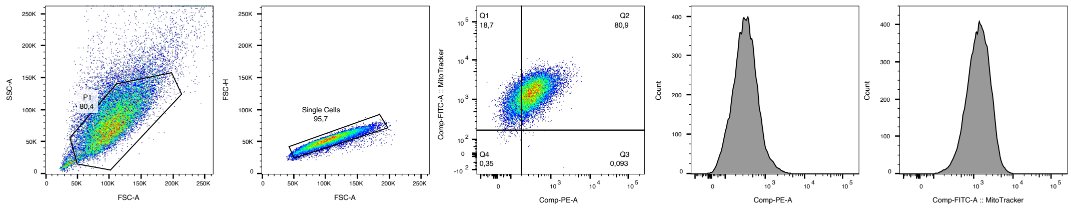

HC402-05A_CMV_Double stained_008.fcs
Ungated
23791

HC402-05A_CMV_Double stained_008.fcs
P1
19135

HC402-05A_CMV_Double stained_008.fcs
Single Cells
18303

HC402-05A_CMV_Double stained_008.fcs
Single Cells
18303
Single Cells : Mean : Comp-PE-A: 429

HC402-05A_CMV_Double stained_008.fcs
Single Cells : Mean : MitoTracker: 1607
Single Cells
18303

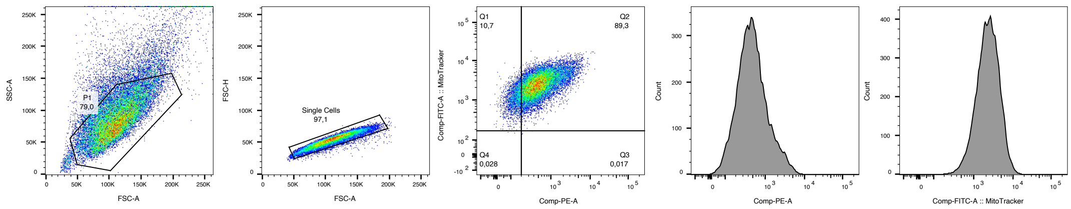

HC402-05A_WT_Double stained_009.fcs
Ungated
23319

HC402-05A_WT_Double stained_009.fcs
P1
18422

HC402-05A_WT_Double stained_009.fcs
Single Cells
17881

HC402-05A_WT_Double stained_009.fcs
Single Cells
17881
Single Cells : Mean : Comp-PE-A: 732

HC402-05A_WT_Double stained_009.fcs
Single Cells : Mean : MitoTracker: 2772
Single Cells
17881

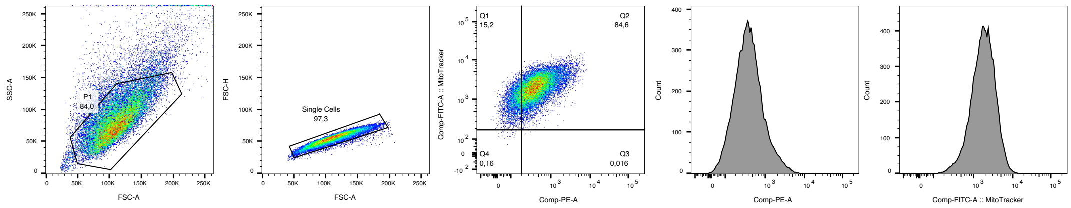

HC402-05A_156_Double stained_010.fcs
Ungated
22875

HC402-05A_156_Double stained_010.fcs
P1
19205

HC402-05A_156_Double stained_010.fcs
Single Cells
18679

HC402-05A_156_Double stained_010.fcs
Single Cells
18679
Single Cells : Mean : Comp-PE-A: 579

HC402-05A_156_Double stained_010.fcs
Single Cells : Mean : MitoTracker: 2164
Single Cells
18679

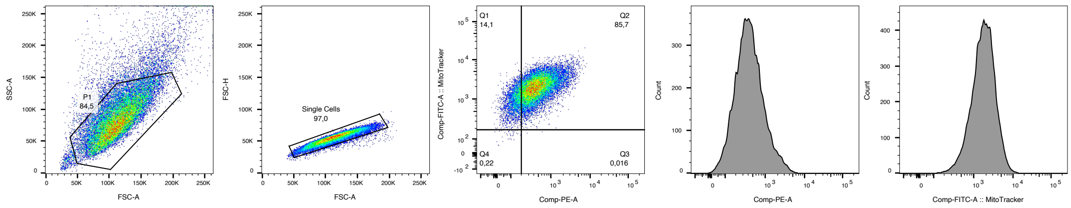

HC402-05A_248_Double stained_011.fcs
Ungated
22973

HC402-05A_248_Double stained_011.fcs
P1
19412

HC402-05A_248_Double stained_011.fcs
Single Cells
18822

HC402-05A_248_Double stained_011.fcs
Single Cells
18822
Single Cells : Mean : Comp-PE-A: 585

HC402-05A_248_Double stained_011.fcs
Single Cells : Mean : MitoTracker: 2143
Single Cells
18822

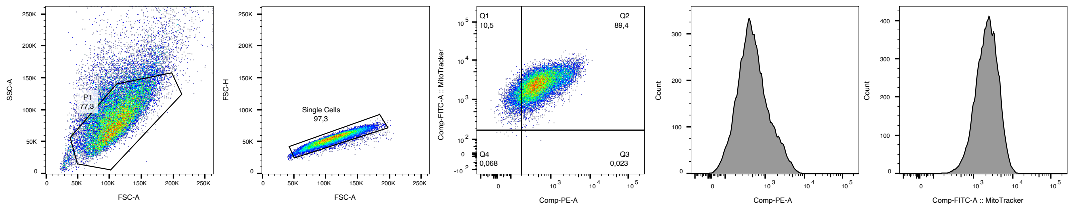

HC402-05A_327_Double stained_012.fcs
Ungated
23466

HC402-05A_327_Double stained_012.fcs
P1
18131

HC402-05A_327_Double stained_012.fcs
Single Cells
17648

HC402-05A_327_Double stained_012.fcs
Single Cells
17648
Single Cells : Mean : Comp-PE-A: 778

HC402-05A_327_Double stained_012.fcs
Single Cells : Mean : MitoTracker: 2633
Single Cells
17648

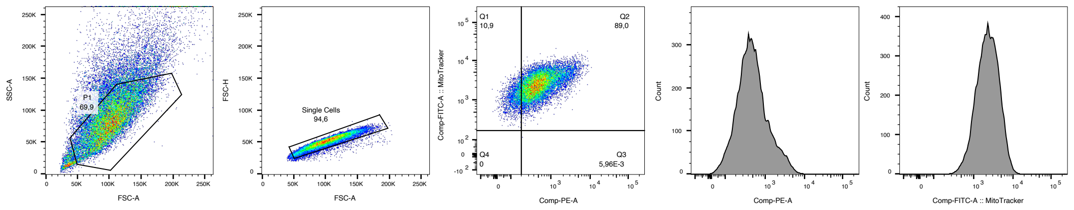

HC402-05A_342_Double stained_013.fcs
Ungated
25386

HC402-05A_342_Double stained_013.fcs
P1
17733

HC402-05A_342_Double stained_013.fcs
Single Cells
16780

HC402-05A_342_Double stained_013.fcs
Single Cells
16780
Single Cells : Mean : Comp-PE-A: 749

HC402-05A_342_Double stained_013.fcs
Single Cells : Mean : MitoTracker: 2736
Single Cells
16780

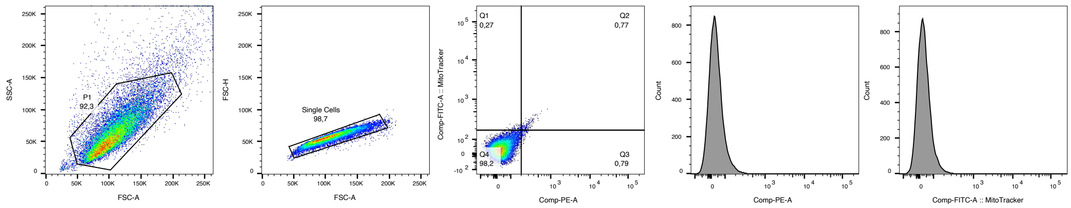

HC402-05A_Unstained_001.fcs
Ungated
21613

HC402-05A_Unstained_001.fcs
P1
19957

HC402-05A_Unstained_001.fcs
Single Cells
19694

HC402-05A_Unstained_001.fcs
Single Cells
19694
Single Cells : Mean : Comp-PE-A: 44,2

HC402-05A_Unstained_001.fcs
Single Cells : Mean : MitoTracker: 40,9
Single Cells
19694

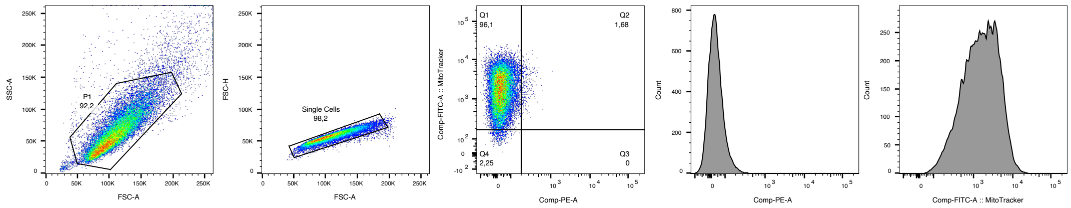

HC402-05A_Mitotracker Green_002.fcs
Ungated
21959

HC402-05A_Mitotracker Green_002.fcs
P1
20251

HC402-05A_Mitotracker Green_002.fcs
Single Cells
19895

HC402-05A_Mitotracker Green_002.fcs
Single Cells
19895
Single Cells : Mean : Comp-PE-A: 43,6

HC402-05A_Mitotracker Green_002.fcs
Single Cells : Mean : MitoTracker: 2343
Single Cells
19895

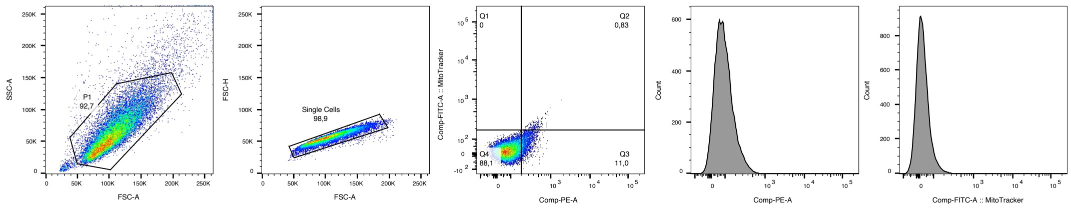

HC402-05A_MitoSOX Red_003.fcs
Ungated
21530

HC402-05A_MitoSOX Red_003.fcs
P1
19952

HC402-05A_MitoSOX Red_003.fcs
Single Cells
19725

HC402-05A_MitoSOX Red_003.fcs
Single Cells
19725
Single Cells : Mean : Comp-PE-A: 108

HC402-05A_MitoSOX Red_003.fcs
Single Cells : Mean : MitoTracker: 32,9
Single Cells
19725

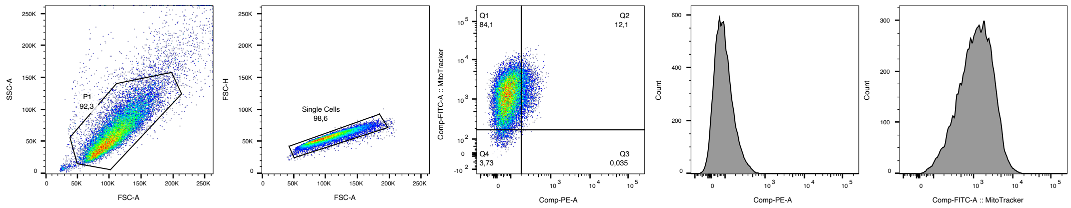

HC402-05A_Double stained_004.fcs
Ungated
21735

HC402-05A_Double stained_004.fcs
P1
20064

HC402-05A_Double stained_004.fcs
Single Cells
19781

HC402-05A_Double stained_004.fcs
Single Cells
19781
Single Cells : Mean : Comp-PE-A: 107

HC402-05A_Double stained_004.fcs
Single Cells : Mean : MitoTracker: 1694
Single Cells
19781

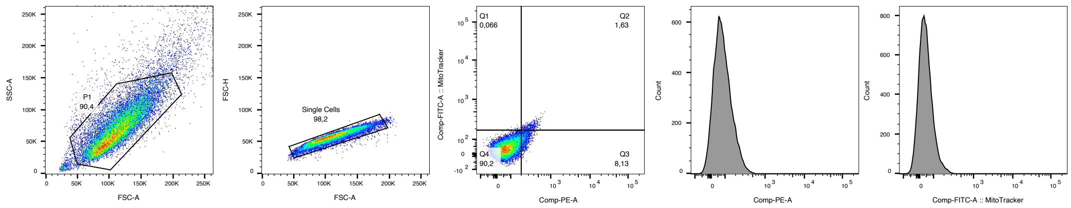

HC402-05A_CMV_Unstained_005.fcs
Ungated
22126

HC402-05A_CMV_Unstained_005.fcs
P1
20010

HC402-05A_CMV_Unstained_005.fcs
Single Cells
19640

HC402-05A_CMV_Unstained_005.fcs
Single Cells
19640
Single Cells : Mean : Comp-PE-A: 96,2

HC402-05A_CMV_Unstained_005.fcs
Single Cells : Mean : MitoTracker: 52,6
Single Cells
19640

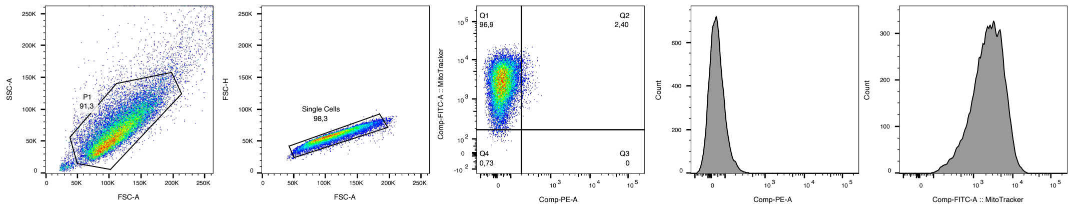

HC402-05A_CMV_Mitotracker Green_006.fcs
Ungated
21963

HC402-05A_CMV_Mitotracker Green_006.fcs
P1
20054

HC402-05A_CMV_Mitotracker Green_006.fcs
Single Cells
19711

HC402-05A_CMV_Mitotracker Green_006.fcs
Single Cells
19711
Single Cells : Mean : Comp-PE-A: 52,2

HC402-05A_CMV_Mitotracker Green_006.fcs
Single Cells : Mean : MitoTracker: 3240
Single Cells
19711

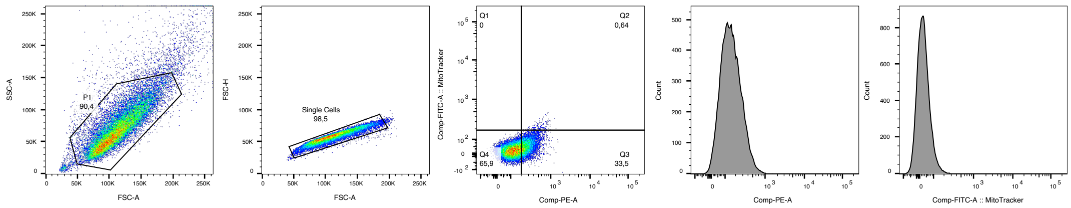

HC402-05A_CMV_MitoSOX Red_007.fcs
Ungated
22030

HC402-05A_CMV_MitoSOX Red_007.fcs
P1
19917

HC402-05A_CMV_MitoSOX Red_007.fcs
Single Cells
19616

HC402-05A_CMV_MitoSOX Red_007.fcs
Single Cells
19616
Single Cells : Mean : Comp-PE-A: 178

HC402-05A_CMV_MitoSOX Red_007.fcs
Single Cells : Mean : MitoTracker: 37,3
Single Cells
19616

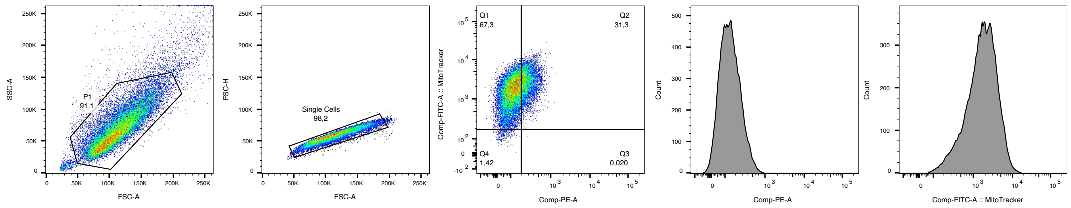

HC402-05A_CMV_Double stained_008.fcs
Ungated
22032

HC402-05A_CMV_Double stained_008.fcs
P1
20063

HC402-05A_CMV_Double stained_008.fcs
Single Cells
19708

HC402-05A_CMV_Double stained_008.fcs
Single Cells
19708
Single Cells : Mean : Comp-PE-A: 168

HC402-05A_CMV_Double stained_008.fcs
Single Cells : Mean : MitoTracker: 2239
Single Cells
19708

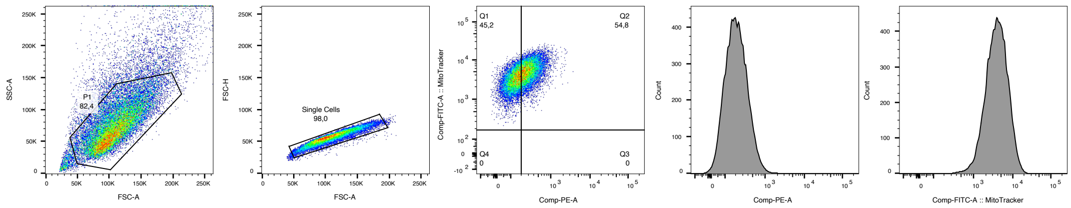

HC402-05A_WT_Double stained_009.fcs
Ungated
23131

HC402-05A_WT_Double stained_009.fcs
P1
19070

HC402-05A_WT_Double stained_009.fcs
Single Cells
18691

HC402-05A_WT_Double stained_009.fcs
Single Cells
18691
Single Cells : Mean : Comp-PE-A: 250

HC402-05A_WT_Double stained_009.fcs
Single Cells : Mean : MitoTracker: 4660
Single Cells
18691

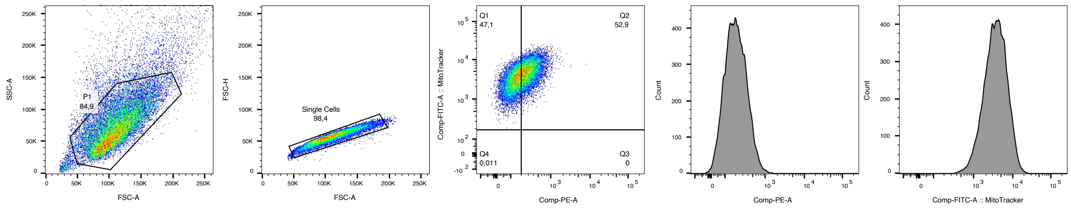

HC402-05A_156_Double stained_010.fcs
Ungated
22523

HC402-05A_156_Double stained_010.fcs
P1
19133

HC402-05A_156_Double stained_010.fcs
Single Cells
18819

HC402-05A_156_Double stained_010.fcs
Single Cells
18819
Single Cells : Mean : Comp-PE-A: 241

HC402-05A_156_Double stained_010.fcs
Single Cells : Mean : MitoTracker: 4402
Single Cells
18819

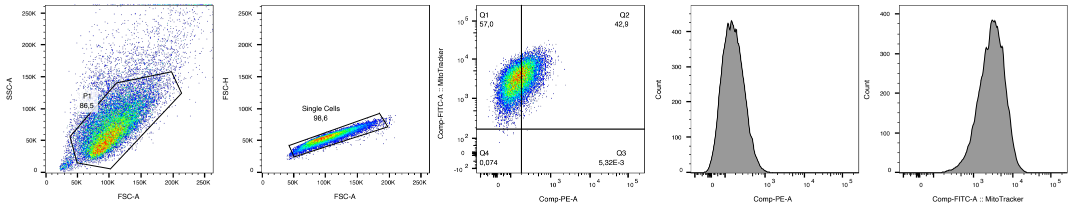

HC402-05A_248_Double stained_011.fcs
Ungated
22035

HC402-05A_248_Double stained_011.fcs
P1
19065

HC402-05A_248_Double stained_011.fcs
Single Cells
18801

HC402-05A_248_Double stained_011.fcs
Single Cells
18801
Single Cells : Mean : Comp-PE-A: 206

HC402-05A_248_Double stained_011.fcs
Single Cells : Mean : MitoTracker: 3671
Single Cells
18801

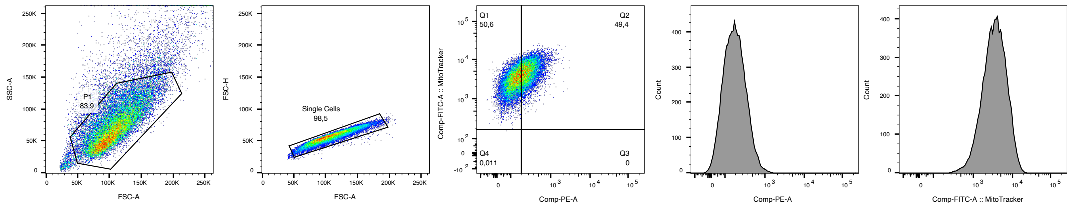

HC402-05A_327_Double stained_012.fcs
Ungated
22743

HC402-05A_327_Double stained_012.fcs
P1
19071

HC402-05A_327_Double stained_012.fcs
Single Cells
18785

HC402-05A_327_Double stained_012.fcs
Single Cells
18785
Single Cells : Mean : Comp-PE-A: 228

HC402-05A_327_Double stained_012.fcs
Single Cells : Mean : MitoTracker: 4247
Single Cells
18785

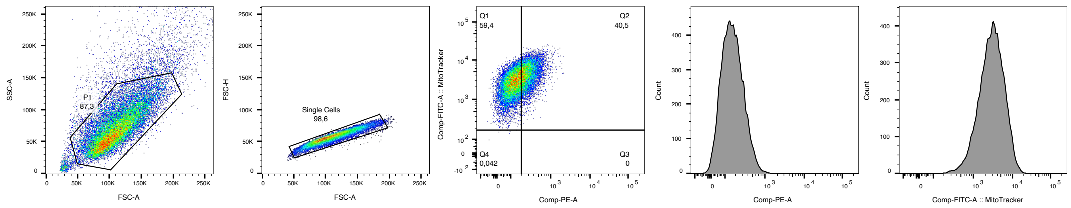

HC402-05A_342_Double stained_013.fcs
Ungated
22376

HC402-05A_342_Double stained_013.fcs
P1
19529

HC402-05A_342_Double stained_013.fcs
Single Cells
19265

HC402-05A_342_Double stained_013.fcs
Single Cells
19265
Single Cells : Mean : Comp-PE-A: 199

HC402-05A_342_Double stained_013.fcs
Single Cells : Mean : MitoTracker: 3650
Single Cells
19265

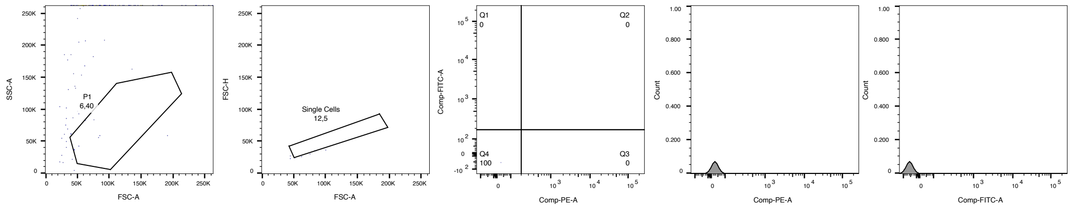

wash_facs clean_014.fcs
Ungated
125

wash_facs clean_014.fcs
P1
8,00

wash_facs clean_014.fcs
Single Cells
1,00

wash_facs clean_014.fcs
Single Cells
1,00
Single Cells : Mean : Comp-PE-A: 35,6

wash_facs clean_014.fcs
Single Cells : Mean : MitoTracker: -53,2
Single Cells
1,00

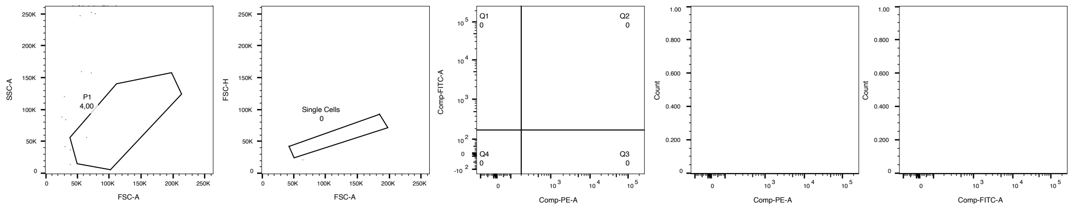

wash_Tube_002_015.fcs
Ungated
25,0

wash_Tube_002_015.fcs
P1
1,00

wash_Tube_002_015.fcs
Single Cells
0

wash_Tube_002_015.fcs
Single Cells
0
Single Cells : Mean : Comp-PE-A: n/a

wash_Tube_002_015.fcs
Single Cells : Mean : MitoTracker: n/a
Single Cells
0

Supplementary Figure S4

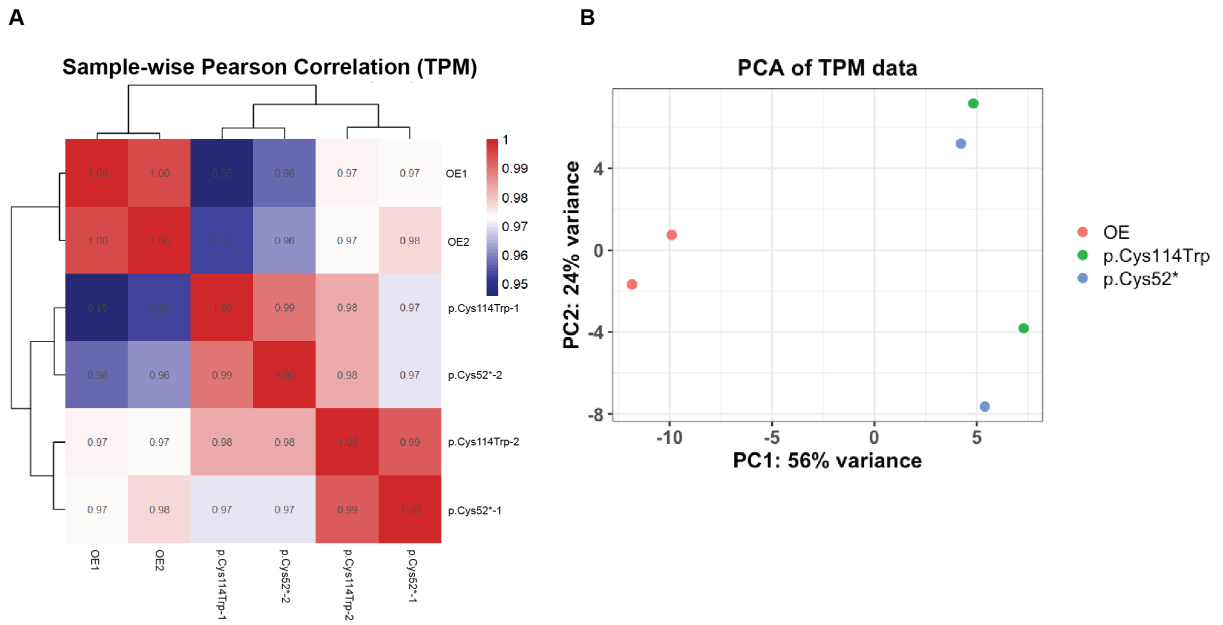

Supplementary Figure S4. Transcriptomic similarity and variance among experimental groups. **(A)** Heatmap showing sample-wise Pearson correlation coefficients based on TPM (Transcripts Per Million) values across all detected transcripts. Each square represents the correlation between a pair of samples, with hierarchical clustering highlighting transcriptional similarity. **(B)** Principal component analysis (PCA) of TPM-normalized expression data. Each point represents a biological replicate, color-coded by experimental group: OE (red), p.Cys114Trp (green), and p.Cys52* (blue). The first two principal components explain 56% and 21.4% of the total variance, respectively. Distinct clustering indicates transcriptomic divergence across conditions.

Supplementary Figure S5

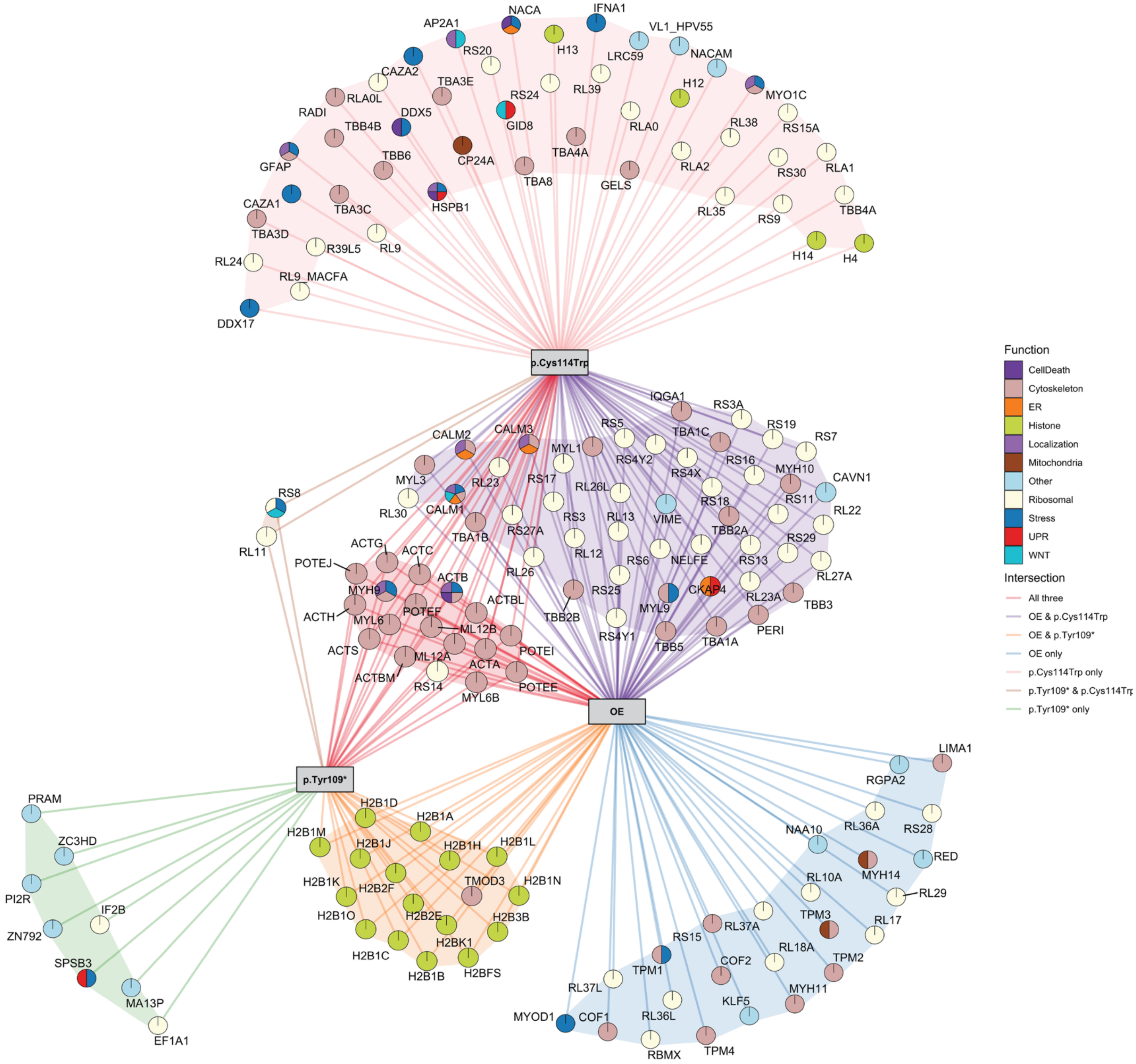

Supplementary Figure S5. Expanded version of the protein–protein interaction (PPI) network derived from LC–MS/MS–based co-immunoprecipitation experiments. HA-tagged WISP3 constructs (OE, p.Cys114Trp, p.Tyr109*) were used to identify interacting proteins in transfected human chondrocytes. Nodes represent unique interactors, grouped by condition-specific or shared interactions, while edge connections indicate the associated construct(s). Functional categorization of proteins is indicated by node color (e.g., cytoskeletal, mitochondrial, ER-associated, ribosomal, stress-related), as defined in the legend. This comprehensive network visualization highlights the variant-specific remodeling of WISP3-associated protein interactions, with p.Cys114Trp showing the most divergent interactome profile compared to OE.
